# Metal–Organic Frameworks–Based Memristors: Materials, Devices, and Applications

**DOI:** 10.3390/molecules27248888

**Published:** 2022-12-14

**Authors:** Fan Shu, Xinhui Chen, Zhe Yu, Pingqi Gao, Gang Liu

**Affiliations:** 1 Department of Micro/Nano Electronics, School of Electronic Information and Electrical Engineering, Shanghai Jiao Tong University, Shanghai 200240, China; 2 School of Chemistry and Chemical Engineering, Shanghai Jiao Tong University, Shanghai 200240, China; 3 College of Information Engineering, Jinhua Polytechnic, Jinhua 321017, China; 4 School of Materials, Sun Yat-sen University, Guangzhou 510275, China

**Keywords:** metal–organic frameworks, memristor, data storage, neuromorphic computing

## Abstract

Facing the explosive growth of data, a number of new micro-nano devices with simple structure, low power consumption, and size scalability have emerged in recent years, such as neuromorphic computing based on memristor. The selection of resistive switching layer materials is extremely important for fabricating of high performance memristors. As an organic-inorganic hybrid material, metal-organic frameworks (MOFs) have the advantages of both inorganic and organic materials, which makes the memristors using it as a resistive switching layer show the characteristics of fast erasing speed, outstanding cycling stability, conspicuous mechanical flexibility, good biocompatibility, etc. Herein, the recent advances of MOFs-based memristors in materials, devices, and applications are summarized, especially the potential applications of MOFs-based memristors in data storage and neuromorphic computing. There also are discussions and analyses of the challenges of the current research to provide valuable insights for the development of MOFs-based memristors.

## 1. Introduction

The rapid development of cloud computing, integrated circuits, and 5G networks has driven derivative applications, such as autonomous driving, the Internet of Things, and virtual reality, but also brings a challenge of how to store and compute the massive data [1,2]. According to the report by International Data Corporation, a world-renowned data company, the annual data generated worldwide will increase from 64 ZB in 2021 to 175 ZB in 2025 [3]. To the traditional von Neumann computing architecture that separates the storage and computation, the data will be frequently transmitted and visited between memory and processors during processing the massive data, which leading to a serious energy consumption [4]. The computing power and efficiency of traditional devices depend on the density of the integrated circuits (IC). However, as the minimum fabrication size of complementary metal oxide semiconductor (CMOS) devices gradually approaches the physical limit, the development of IC falls into the bottleneck period of Moore’s Law [5]. It means that the “storage wall” and “bandwidth wall” of the von Neumann architecture make it unable to cope with the challenge from massive data [6,7]. There is an urgent need to develop a series of post-Moore era’s storage and computing devices, which has simple structure, low power consumption, and size scalability [8].

As one of the candidates for high-performance post-Moore devices, memristors have not only been systematically studied in terms of mechanism principles, fabrication technology, neural networks, circuit design, etc., but also have a certain market share in commercial applications of memory, hardware security, computers, etc. Taking memory as an example, the market forecasts memristive memory to reach USD 5.6 billion by 2026, accounting for 2% of the market share. Memristor is also called resistive random-access memory (RRAM) [9], which represents the relationship of magnetic flux to electric charge [10]. It is a non-volatile two-terminal device with simple structure [11,12,13], i.e., electrode/resistive switching layer/electrode sandwich stack. When the voltage applied between the two electrodes reaches the set voltage and reset voltage, respectively, the resistance value of the switching layer will reversibly switch between a high resistance state (HRS) and a low resistance state (LRS) [14,15], which is several orders of magnitude smaller than HRS. Importantly, lower applied voltages enable memristors to have a characteristic of low power consumption [16,17,18]. Meanwhile, the fabrication process of memristors is compatible with CMOS process technology [19], allowing it to be large cross-bar arrays easily and exhibit a size scalability. More excellent properties of memristors can be realized by changing or adjusting the materials of resistive switching layer [20,21,22,23,24,25].

**Figure 1:**
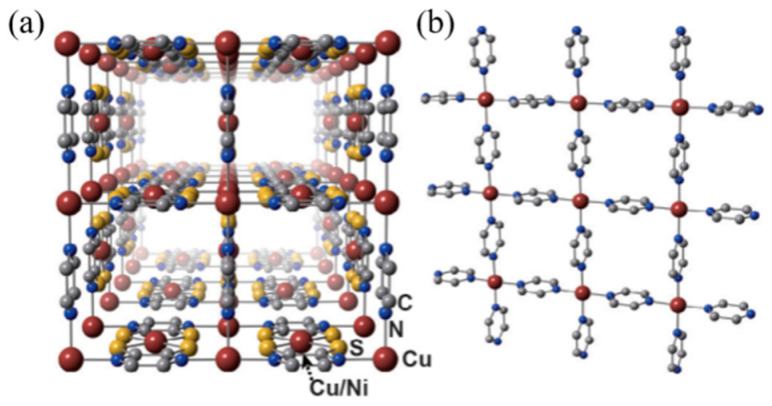
(**a**) the structure of conductive MOFs, Cu[Cu(pdt)_2_] and Cu[Ni(pdt)_2_]. Reprinted with permission [32] from Wiley. (**b**) single Cu(pyrazine) in Cu[Ni(pdt)_2_], which is connected by Ni(dithiolene) units. Reprinted with permission [33] from Wiley.

Presently, the reported resistive switching materials can be roughly divided into inorganic resistive switching materials and organic resistive switching materials, and each has its own advantages [26]. For example, memristors based on inorganic resistive switching materials represented by metal oxides have the advantages of high switching ratio, excellent cycle stability, and fast inversion speed; memristors based on organic resistive switching materials have the advantages of mechanical flexibility, biocompatibility, and decomposability [27]. As an organic-inorganic hybrid materials (Figure 1a), MOFs are self-assembled by organic ligands and inorganic metal ions or clusters relying on coordination bonds (Figure 1b), so that they can combine the advantages of inorganic materials and organic materials well to develop MOFs-based memristors with better performance. The emergence of several toolkit extensions for synthesizing MOFs benefits from the diversity and tunability of organic molecular structures and metal ion species, which provides a good theoretical guide for the experimental protocol and design of MOFs with specific structures and pore sizes [28]. A research roadmap of MOFs for electronic exploration was first proposed in 2011 and updated and in 2017 [29]. It highlighted the important of MOFs as the component of solid-state electronics [30], stimulating researchers to investigate their potential applications. There are many breakthroughs in the potential applications of MOFs in electronics, even though the discussed MOFs have been strictly restricted to exclude the most of coordination polymers [31]. It served as a guide to lead the community to discuss the possibilities of MOFs in virtually all major application areas, including sensors, FETs, electrical characterization, storage, and neuromorphic computing.

In commercial applications, MOFs have not yet been used in electronic devices, but there are many conceptual devices about it have been validated [34,35,36], especially MOFs-based memristors have been reported to exhibit excellent low power consumption, resistive state regulation, and long-term stability. More and more studies focus on the performance improvement of device and the exploration of underlying mechanism [37]. These fundamental properties are the essence of enabling potential applications. For example, the synaptic function of MOFs-based memristors is necessary to enable neuromorphic computing, which can be achieved by applying electric fields or light. The reported flexible MOFs-based memristors also show an irreplaceable advantage in wearable devices. Organic or inorganic memristors have been systematically researched and preliminarily commercialized, so we believe that MOFs-based memristors with high performance will make faster progress on this basis.

**Figure 2:**
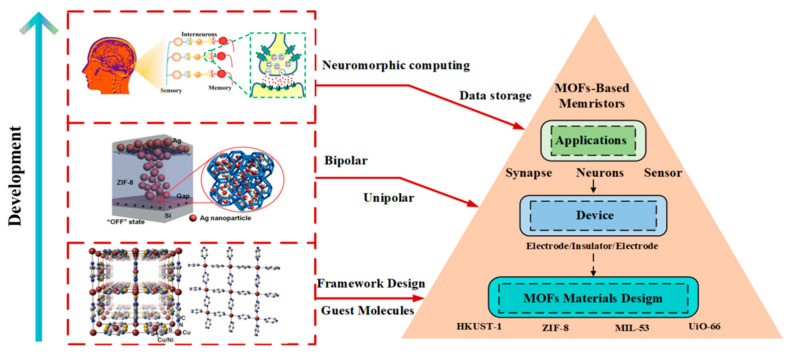
The architecture diagram of this review.

In this review, we focus on the development of MOFs-based memristors in the past two decades, summarize and discuss the properties and fabrication of materials, structure and electrical parameters of devices, and resistive switching mechanism (Figure 2). According to the latest reported progress of MOFs electrical properties and memristor applications, the two main potential applications of data storage and neuromorphic computing are deeply analyzed. Finally, we try to discuss the challenges and opportunities encountered from the three levels of fabrication process, device, and application. We hope that the outlook of future research and development of MOFs-based memristors will facilitate their development in storage and energy-efficient neuromorphic computing applications.

## 2. MOFs-Based Memristors

Limited by the low repeatability and high formation voltage of traditional metal oxide memristors [38,39], many new functional materials (perovskites [40], 2D materials [41,42], biomaterials [43], polyoxometalates [44], organic materials [45,46], etc.) have been explored for memristor applications. Among them, the research of MOFs-based memristor is still in the preliminary stage because of the absence of a high-performance MOF series and a clear development direction. At the same time, it is generally believed that insulating materials with high porosity are easy to short circuit and not suitable for electronic devices. However, the framework of MOFs not only provides a variety of conduction mechanisms (organic conduction, chemical bonds conduction, space conduction of π conjugation, etc.), but also bring some unique properties. For instances, the guest molecules can fill the holes left by the skeleton to improve the overall conductivity of the material; the regular porous skeleton with limited pore size provides a smooth path for the selective migration and penetration of ions. Therefore, it has good application prospects in RRAM, flexible electronic devices, photovoltaic, sensors, and other fields for MOF materials.

At present, people have adopted a series of methods to modify MOF materials by utilizing their unique characteristics of structure, conductive mechanism, design and synthesis, resulting in that the MOF materials play an important role in the field of memristor. It is particularly important to comprehensively introduce the development of MOFs-based memristors. Table 1 lists some performance parameters of MOFs-based memristors and others memristors in order to exhibit the superiority of MOFs materials.

### 2.1. Materials Properties 

A key requirement for the design and manufacture of electronic devices containing MOFs is to understand their fundamental charge transport laws. Compared with silicon and organic semiconductor materials, little is known about the performance of MOFs. 

MOFs are porous network structure crystals composed of metal ions or metal clusters and organic ligands. Its skeleton is made up of organic ligands, which leads to low conductivity of general materials [57]. It is well known that crystals with a high degree of order and almost no defects will provide an effective transport pathway for charged particles [58]. Therefore, there are many ways to improve the conductivity of MOFs. For example: (1) Modification and optimization of organic linkers for assembling frameworks; (2) Using the periodic pore array inside the MOFs material to achieve ion penetration, capture and transport in the supramolecular network; (3) The use of organic semiconductors to fill voids or introduce appropriate host molecules can also help to change the electrical properties of the host material, thereby allowing ion and electron transport [59]. 

In the following, we will introduce the conductive mechanism of MOFs and other available basic properties from multiple perspectives.

**Figure 3:**
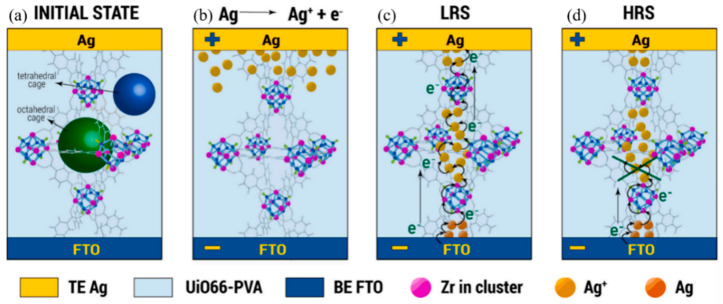
(**a**) the octahedral space and initial state within the UiO-66. (**b**) the permeation of Ag^+^ on the electrode surface during the application of positive voltage. (**c**) schematic diagram of silver conduction path with complete conduction in low resistance state. (**d**) the state in which the conductive path is broken in the state of high resistance. Reprinted from [60] with permission from Elsevier.

Under sufficient external stimuli, the active electrons generated in the molecule can hop to the electron-deficient region along the chemical bond (coordination bond, etc.) between the metal and the linker in the MOFs. However, it requires a certain amount of cations to penetrate between the pores, and enough energy for electron to hop the band gap, this has certain requirements for the stability of the molecule itself under applied energy. Among the MOFs materials, UiOs (UiO is the abbreviation of Oslo University) material is composed of a Zr-containing octahedral [Zr6O4(OH)4] metal cluster core, which is connected to 12 aromatic molecules containing para-dicarboxyl groups, such as terephthalic acid (BDC), to form a series of three-dimensional microporous structure materials containing octahedral central pore cages and eight tetrahedral corner cages. Because of its high coordination number, the material has superior heat resistance, acid resistance and alkali resistance stability [42]. In UiO-66 fabricated by Tran et al., they found that when a sufficient positive voltage is applied, The Ag^+^ generated by the electrode discharge accepts electrons that jump through the metal junction Zr6 and is reduced in the UiO-66 cavity to form silver nanoparticles [60]. The high porosity of materials and penetrability of Ag^+^ between electrodes enable the Ag^+^ to be continuously reduced, causing the silver nanoparticles to splice together to form a weakly bound vertically conductive channel inside the almost insulating UiO-66. The whole process is shown in Figure 3a–d. Naturally, the thickness of the silver conductive wire is closely related to the applied voltage in this process, but a thicker conductive wire also means a weaker cycle.

Electrical conductivity through the space is very rare in MOFs, such as equistructural metals-organic framework M_2_(TTFTB) (M = Mn, Co, Zn, Cd; H_4_TTFTB = tetrathiafulvalene tetrabenzoates) [61], and highly conjugated phenanthrene molecular sieve NNU-27 having a long-range π-stacked zigzag chain form. Electrons can be sterically conducted from the stacking direction through π stacking [62]. Although both are conductive through space charge transport, the conductivity of NNU-27 is five times than that of M_2_(TTFTB) due to the presence of a more conjugated long-range π-conjugated arrangement in NNU-27, which is a key factor in enhancing charge mobility between aromatic molecules. The conjugations in M_2_(TTFTB) and in NNU-27 are both arranged along the *c*-axis of the crystal, whereas their ligand planes are perpendicular and parallel to the *c*-axis, respectively. The space charge transport in M_2_(TTFTB) is carried out in a ‘spiral ladder’ manner, while the conductive path of NNU-27 is a zigzag chain to have a wider π electron overlap region, so that its charge transport path is more conjugated than that in M_2_(TTFTB).

Compared with the other two conduction methods, it has received more research for the charge transport mechanism established by guest molecules. PCNs (Pocket-Channel Frameworks) series MOFs materials contain multiple cubic octahedral nanocage pores, which have great potential in the field of gas adsorption due to their outstanding specific surface area and adsorption properties [63]. HKUST-1 is a classical PCN material composed of Cu ions and 1,3,5-benzenetricarboxylate (BTC) ligands in a cubic lattice [64]. Under the synergistic effect of regular porosity, the appropriate interaction between the adsorbed guest molecules and the skeleton contributes greatly to the improvement of conductivity. A classic example is the introduction of different amounts of tetracyanoquinodimethane (TCNQ) into the pores of HKUST-1, which can control the conductivity of the material by six orders of magnitude [58]. Wang et al. also found that loading ferrocene on HKUST-1 can increase the overall electron density by conjugating molecules on the framework. It can help some iron ions that are loosely bound to obtain a better penetration/movement performance in molecular sieves, leading to a greatly improvement for the switching performance of device [59].

ZIFs (Zeolitic Imidazolate Frameworks) are another type of MOF materials with good adsorption capacity and have a zeolite-like structure [65]. ZIF-8, which often appears in this paper, is self-assembled by Zn and N on dimethylimidazole in a four-coordinated manner. In view of its permanent pore properties and easy synthesis, it also has certain advantages in adsorbing gas.

**Figure 4:**
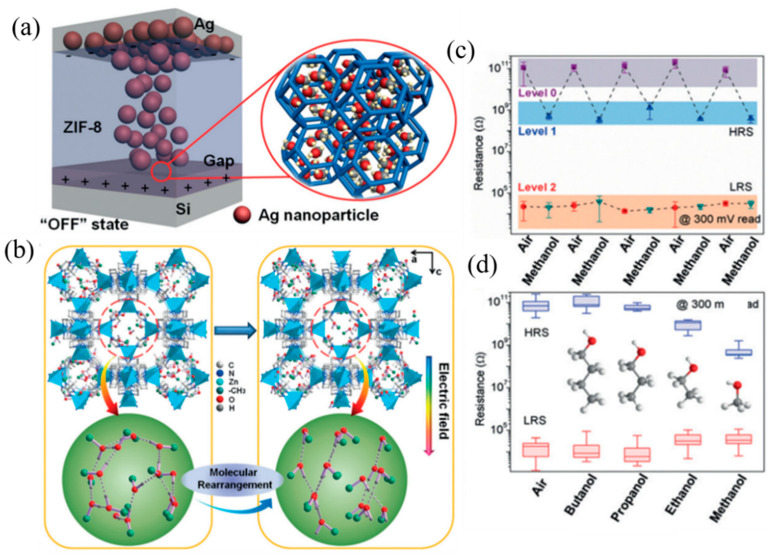
(**a**) Schematic diagram of the ZIF-8-based device in the OFF state. Methanol was adsorbed in ZIF-8 crystals. (**b**) Molecular dynamics simulation scheme and statistical analysis of the adsorption of alcohol molecules in ZIF-8 crystals, with or without an applied electric field (right and left of the figure, respectively), the packing mode of methanol molecules in ZIF-8 crystals. (**c**) Repeated resistive switching cycles of the device operating in air and saturated methanol vapor at a read voltage of 300 mV. (**d**) Statistical analysis of two resistance states for different saturated alcohol vapors at room temperature 300 mV readout voltage. Reprinted from [49] with permission from Wiley.

Liu et al. made memristors with high chemical and thermal stability using ZIF-8 (zeolite imidazole skeleton-8) material [49]. Based on the pore properties of ZIF permanent pores and the adsorption of small molecules by host-guest interaction, the resistive switching characteristics of ZIF can be chemically modulated. The thermal resistance of the device will gradually decrease to another stable thermal resistance state in methanol vapor, and the thermal resistance change curve accords with the methanol adsorption isotherm of ZIF-8 molecular sieve. The simulation results show that before pressurizing the ZIF-8 crystal, the adsorbed methanol molecules are arranged in a relatively free packing mode in the ZIF-8 crystal cage, and the angle between the hydrogen bond and the *c*-axis is irregularly distributed (Figure 4b). By applying an electric field in the *c*-axis direction of the ZIF-8 single crystal, the methanol molecules can be arranged in an orderly manner, and its dipoles are basically arranged along the direction of the external electric field (Figure 4a,b). When the operating condition of the device changes from air to saturated methanol vapor, the HRS value of the ZIF-8-based memory decreases sharply, and the corresponding resistance turn-off ratio decreases from 10^7^ to 10^4^. At the same time, the test shows that the adsorption of other types of alcohols can also show a similar changing trend, and there is a more obvious change in fewer carbon chains. It may be related to the number of adsorbed molecules, polarity, and other factors (Figure 4c,d).

**Figure 5:**
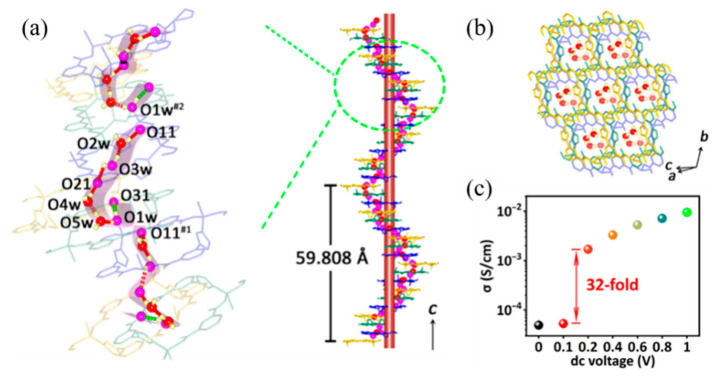
(**a**) Three-dimensional stack for hydrogen bonding chain fragment along the *c*-axis. (**b**) Lattice water molecules located in hexagonal nanochannels of FJU-23-H_2_O. (**c**) Conductivity changes of FJU-23-H_2_O under DC voltage. Reprinted from [50] as open-access.

He et al. also used the chemically inert rare earth metal-based MOFs to protect the halogenated metal salts embedded in viologen and chose it as guest molecules to improve photothermal stability [66]. In addition to adsorption and embedding, the interaction between host and guest molecules can also format the new bonds. In other unclassified MOFs, Yao et al. reported a chiral MOFs-based FJU-23-H_2_O, in which several lattice water molecules were filled in the hexagonal nanochannels to form hydrogen bond with the oxygen atom O31 of frameworks (Figure 5a,b) [50], while the *c*-axis direction was not fully connected. When a voltage of 0.2 V was applied to the single crystal, the conductivity of FJU-23-H_2_O increases sharply by a factor of 32 as the guest water molecule turns to form a new hydrogen bond with O11 of the linker (Figure 5c). At the same time, the conduction between the levels is achieved on the *c*-axis, leading to the huge change in the conductivity. This process has been experimentally proven to be reversible.

**Figure 6:**
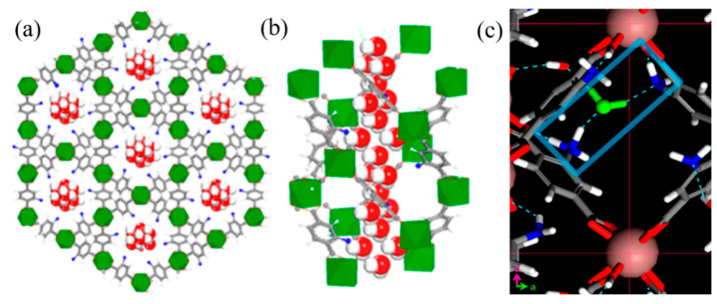
(**a**) Structures along the *c*-axis, and (**b**) along the b-axis by single crystal X-ray diffraction. (**c**) Molecular dynamics simulation snapshot of the spontaneous formation of a stable N···H-O···H-N bridge structure. Water molecules with stable hydrogen bonds are highlighted in green. Color code: IN, pink; N, blue; O, red; H, white. Reprinted from [51] with permission from ACS.

Pan et al. designed an indium MOFs (denoted as RSMOF-1) with the chemical formula [InC_16_H_11_N_2_O_8_]·1.5H_2_O and a twofold-interpenetrated three-dimensional (3D) *β*-quartz topology (Figure 6a). The morphology of the crystals is hexagonal prismatic [51]. The guest water molecules are trapped in the hexagonal helix nanochannels, which has an amine-function-alized wall along the *c*-axis, making the water molecules form a hydrogen-bonding network (Figure 6b). The presence of amino groups in the channels provides abundant sorption sites for water molecules through hydrogen-bonding interactions. From the result of first-principles MD simulation, the formation of N···H-O···H-N bridge structures will occur in random directions without the effective regulation of external electric fields, so that RSMOF-1 is characterized by intrinsic nonpolarity (Figure 6c). However, an external electric field may force the N···H-O···H-N bridge structure to flip and get aligned along the external field direction, resulting in an order-disorder-type ferroelectric polarization of RSMOF-1.

**Figure 7:**
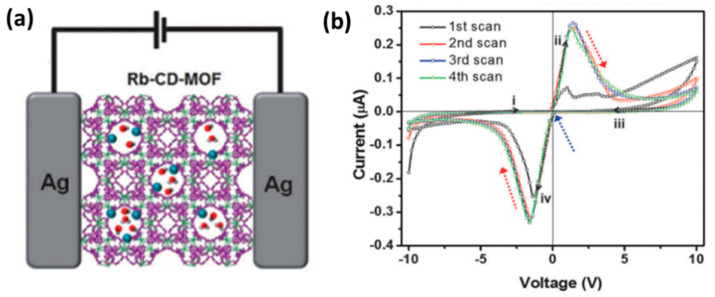
(**a**) A MOF memory device comprising two silver electrodes and Rb-CD-MOF crystal containing hydroxy ions, rubidium ions, and water. (**b**) Cyclic voltammetry analysis of a silver/MOF/silver device heterostructure. Reprinted from [35] with permission from Wiley.

Ionic conduction is an important principle in many energy storage and conversion devices, and is widely used in fuel cells, lithium-ion batteries, and etc. The regularly arranged nanochannels and the limited pore size of MOFs is beneficial to selectively transport ions, which exhibits great advantages in the process of ion penetration and conduction. Yoon et al. used the characteristics of abundant hydroxide ions and small pore size in the synthesized Rb-CD-MOF [35] and used the MOFs to generate and selectively pass OH^−^ to conduct electricity while promoting the redox process on the electrode surface. In the presence of water, RbOH in MOFs is decomposed into Rb^+^ and OH^−^ (Figure 7a), and positive potential will be oxidized at the silver electrode/MOFs interface, where OH^−^ provides an alkaline environment at the interface. Because of the selective passage of MOFs aperture to ions, Ag^+^ only stays at the interface inducing the passivation of the electrode and corresponding electrical characteristics (Figure 7b).

In addition to the selective passage, the porous structure also has a favorable effect on the penetration and transport of ordinary ions, such as the transport of electrode metal ions between layers, which is an important feature of conductive filament mechanisms. It is precisely because of the excellent ion transport properties of MOFs that the electrode metal ions can shuttle between the upper and lower electrodes and form metal conductive wires to connect. More detailed examples and introductions can be found in Section 3.2 Ion penetration.

**Figure 8:**
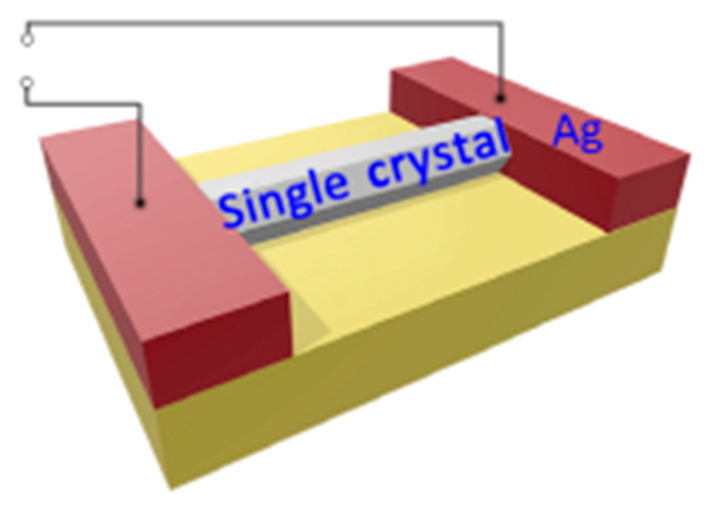
The illustration of electrical performance test. Reprinted from [50] as open-access.

The unique features of MOFs also provide new interesting design ideas for the development of memristors. some reports that the MOFs are directly used as functional layers due to its excellent crystalline property. Yoon et al. took advantage of the characteristic of γ-cyclodextrin-based Rb-CD-MOF that can grow to millimeter-sized single crystals to fabricate devices with high performance, which shows short preparation process of film and economic benefits [35]. Coincidentally, the above-mentioned Yao et al. also prepared single crystals that can be directly used in the fabrication of devices (Figure 8) [50].

**Figure 9:**
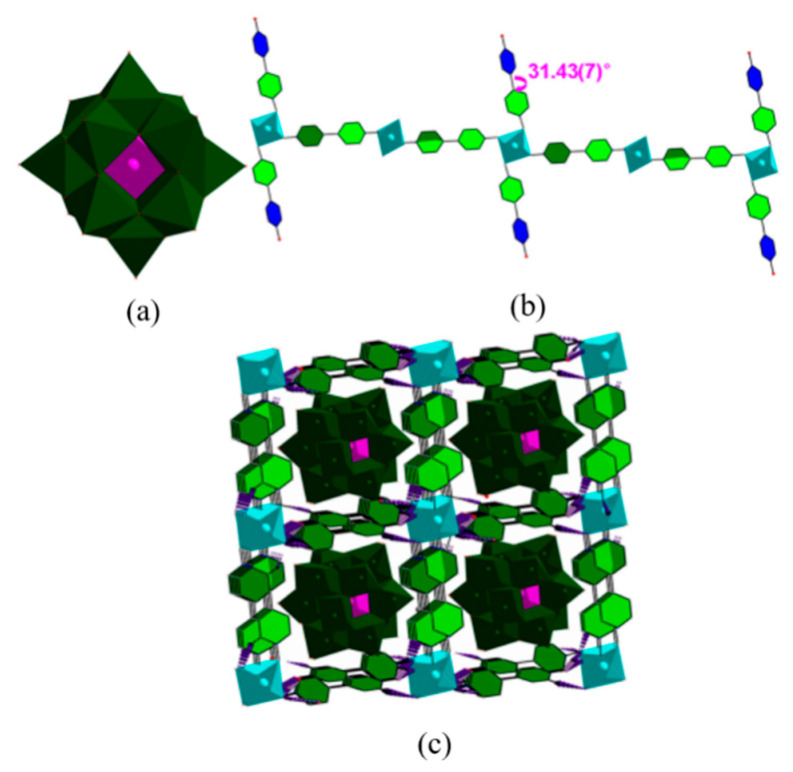
(**a**) Saturated (α-GeW_12_O_40_)^4−^ polyoxometalate anion; (**b**) 1D [Co_2_(bpdo)_4_(H_2_O)_6_]_n_^4n+^ cationic chain; (**c**) diagram of POM@MOF with hydrogen atoms without involving H-bonds being omitted for clarity. Reprinted with permission [67] from ACS.

Due to the high plasticity of MOFs, the original heat-labile materials can also have thermodynamic properties through improvement and modification, such as the replacement of skeleton and the change of energy band. It is reported that utilizing the thermodynamic properties of MOFs to design RRAM with high performance. Chen et al. synthesized a thermochromic polyoxometalate-based MOF, referred to as POMOF for short, with the chemical formula {[Co_2_(bpdo)_4_(H_2_O)_6_](α-GeW_12_O_40_)}·4(H_2_O)}_n_ (α − 1) [67]. In heating conditions, the lattice water is removed, the intramolecular hydrogen bonds are strengthened with decreasing distance, and the organic linker shrinks, but the Kegkin-type POM (α-GeW_12_O_40_)^4−^ anion passes through a large number of C-H…OPOM hydrogens. The bonds are anchored in the metal shellac cation [Co_2_(bpdo)_4_(H_2_O)_6_]_n_^4n+^ so that the high temperature cannot destroy its skeleton (Figure 9a–c). As a result, although this temperature is very dangerous for general organic linkers at high temperature of 150 °C, the functional layer of the device can change color while the memristive properties of device are still maintained, and it can be used to a temperature sensor depending on the color change of functional layer with temperature. Rana et al. used Ag-TCNQ to fabricate a temperature-stimulated RRAM device, which is different from the phase change memory that affects the electrical properties through temperature. The Ag-TCNQ reported by them uses thermal energy in metal-semiconductor Alternation of Schottky barriers at the interface achieves switching performance [68]. A more detailed introduction can be found in the mechanism section below.

### 2.2. Synthesis of Functional MOFs

There are many methods for synthesizing MOFs, and different synthesis methods have a relatively large influence on the quality of the product [52]. We have selected several synthesis methods of MOFs commonly used in memristor research.

#### 2.2.1. Solvothermal Reaction

Solvothermal reaction is a chemical method that the reactants are mixed together and reacted at a certain temperature and pressure. Autoclaves and organic or non-aqueous solvents are usually used as closed systems and solvents, respectively. Relying on the high temperature and high pressure enclosed condition, this method provides a special physical and chemical environment for precursors to be activated in liquid phase or other supercritical conditions, realizing the chemical reactions and crystallization processes that are difficult to occur at normal condition. The process is easy to control the formation of phase and the shape of particle size, and its product also has good dispersibility.

The FJU-23-H_2_O skeleton was synthesized by solvothermal method. Firstly, the monomer zinc nitrate and 5-triazole isophthalic acid were mixed in a mixed solvent of DMF and water. Then, the mixture solution is heated in the reactor to initiate self-assembly. Finally, the crystals were obtained after cooling. The product has three crystallographically independent Zn(II) atoms bridged with three fully deprotonated L^2−^ ligands in the chiral hexagonal space group P6_5_, forming their own single honeycomb sheets. It is worth noting that these three honeycomb sheets are not coincident due to a six-fold helix parallel to the *c*-axis, and the parallel six-layer stack provides a helical arrangement of 18 sheets with a pitch of 59.808 Å, which is the highest-level stack ever observed (Figure 5a) [50]. Similarly, the constituent materials of RSMOF-1 are synthesized [51], including monomers indium nitrate, 2-amino-1,4-phthalic acid, and 1,4-diazepine Hexa[2.2.2]octane, as well as the product and crystal morphology are a double interpenetrating 3D *β*-quartz topology and hexagonal prism respectively.

As the most classic solvothermal reaction, hydrothermal method is also applicable to the synthesis of MOFs. In the report of Chen et al., they used water as a solvent, added various raw materials and reacted at high temperature in a Teflon-lined stainless steel container for several days to obtain their self-made POMOF material [51]. Although this method is simple and convenient, the bulk product may affect the application in electronic devices.

#### 2.2.2. Surfactant-Assisted Method

**Figure 10:**
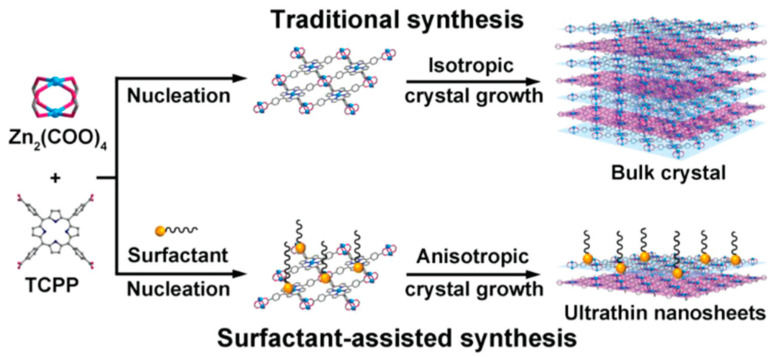
The traditional synthesis and surfactant-assisted synthesis of MOFs. Top: During the synthesis of MOFs in the traditional method, the isotropic growth generates the bulk crystal of MOFs. Bottom: By using developed surfactant-assisted synthetic method, resulting in the formation of ultrathin MOFs nanosheets. Reprinted from [69] with permission from Wiley.

The surfactant molecules selectively adsorb on the surface of MOFs to control the growth of crystal and make them grow into two-dimensional (2D) MOFs nanosheets according to anisotropy, which was first reported in 2009 to synthesize MOFs. Surfactants have been widely used to control the growth of nanocrystals with size and shape, and they play a key role in controlling the growth of MOFs crystals by selectively adsorbing on specific faces of the nanocrystals, leading to anisotropic MOFs (Figure 10). Unlike the solvent method to obtain bulk products, the anisotropic growth method can prepare of ultra-thin MOFs nanosheets, while the traditional solvent method can only obtain bulk products [69].

**Figure 11:**
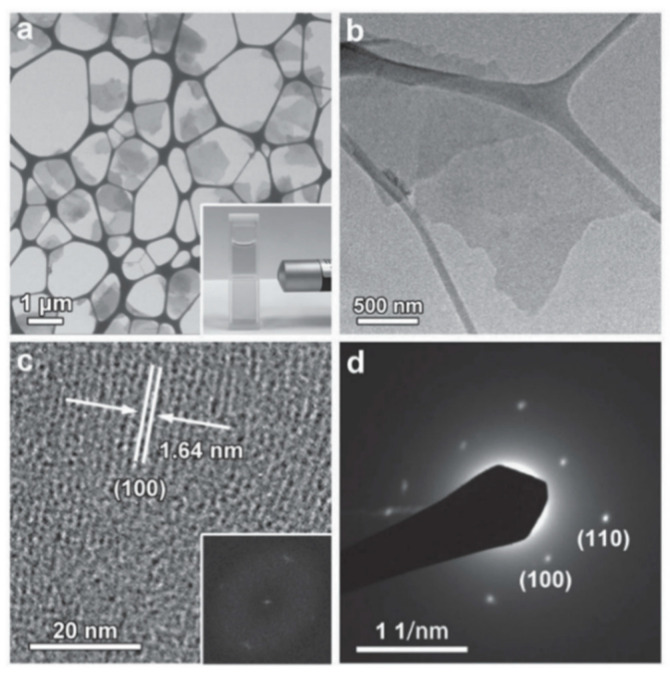
(**a**) STEM image of Zn-TCPP nanosheets obtained by SEM with a transmission electron detector. Inset: Tyndall effect of colloidal Zn-TCPP nanosheet in ethanol. (**b**) TEM image of a single Zn-TCPP nanosheet. (**c**) HRTEM image of Zn-TCPP nanosheet and corresponding FFT patterns (inset). (**d**) SAED pattern of Zn-TCPP nanosheets in (**b**). Reprinted from [69] with permission from Wiley.

In this way, the MOFs material M-TCPP (M is metal Zn, Cu, Cd or Co) with switching properties was synthesized Ding et al. Take Zn-TCPP as an example [53]: Firstly, Zn(NO_3_)_2_·6H_2_O, PVPy and pyrazine were dissolved in a mixed solvent containing DMF and ethanol; then, the mixed solvent of TCPP was added to the above reaction system; lastly, Zn-TCPP nanosheets can be obtained as shown in Figure 11a–d [69]. The nanowires with two-dimensional structure are larger than the bulk than the surface, and have more highly accessible active centers, which is of great significance for electrochemical and sensing applications. It is also proved by comparative tests that the memristor characteristics of the nanosheets are much better than those of the bulk products [53].

#### 2.2.3. Liquid Phase Epitaxy Approach

Liquid phase epitaxy is a method in which solid substances are precipitated from solution and deposited on a substrate to form a single crystal thin layer. It is also called liquid phase self-assembly method, which is the main method for growing compound semiconductor single crystal thin layers in the production of electronic devices.

**Figure 12:**
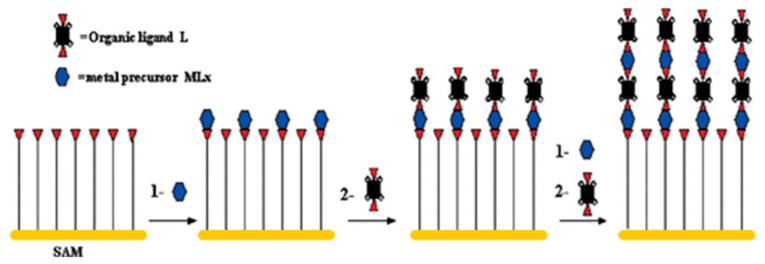
Schematic diagram for the step-by-step growth of the MOFs on the SAM, by repeated immersion cycles, first in solution of metal precursor and subsequently in a solution of organic ligand. Reprinted with permission [54] from ACS.

In this method, a film-like product is grown directly on the surface of the substrate by repeatedly dipping the substrate in solutions of various monomers to initiate chemical reactions on the surface of the substrate (Figure 12). The final product film shows high quality, good morphology consistency, and low root mean square roughness. Meanwhile, its desired thickness can be controlled by the number of substrate wettings [54].

Following this method, Pan and co-workers prepared the memristor with functional layer of HKUST-1 (Cu_3_(BTC)_2_) [70]. The Au-OH substrate was immersed in the ethanol solution of the metal solution Cu(CH_3_COO)_2_, then the solution was removed and the substrate was dried in a nitrogen reactor. Subsequently, the substrate was soaked in the ethanol solution of the ligand benzene-1mine3-tricarboxylic acid (BTC) and then dried again. The above process was repeated 100 times to grow 100 layers of Cu_3_(BTC)_2_ on the substrate. High quality continuous MOFs nanofilms with good resistance switching properties and mechanical flexibility were obtained: film thickness of ~130 nm, particle size of 40–80 nm, and root mean square roughness of ~4 nm.

Yi et al. also adopted this method to synthesize MIL-53, a hydrogen-bond-driven expansion-contraction breathing MOF [55], by sequentially immersing the surface-functionalized composite electrode in a saturated aqueous solution of raw material AlCl_3_ and terephthalic acid (H_2_BDC) for several minutes. Different from the above methods, Yi et al. synthesized the material at a high temperature to drive the self-assembly of two raw materials. The material is eventually attached to the electrode, eliminating the need for membrane formation. Albano et al. also synthesized film-like SURMOF by introducing samples into copper acetate and BTC, which is similar to the method of liquid phase epitaxy [56].

**Figure 13:**
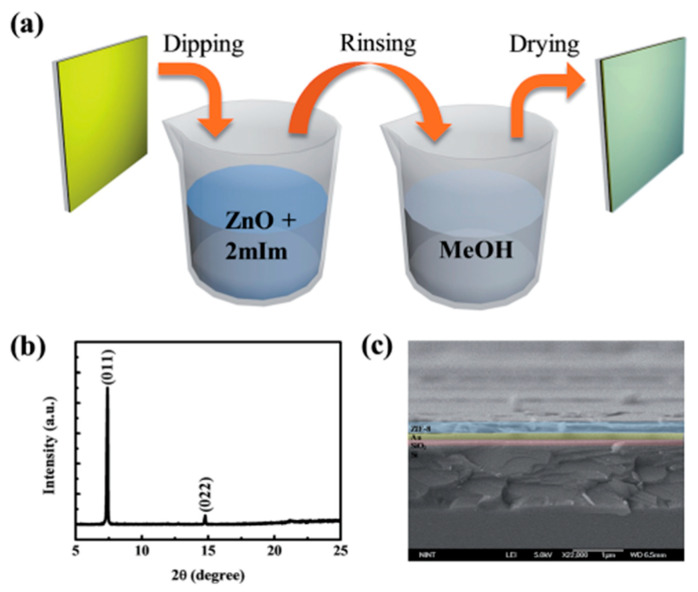
Synthesis of ZIF-8 thin film on the substrate. (**a**) Synthesis method of the ZIF-8 thin film on the gold-coated flexible substrate. (**b**) XRD pattern after coating with ZIF-8 thin film. (**c**) Cross-sectional SEM image of ZIF-8 thin film on the Au bottom layer. Reprinted with permission [71] from RSC.

In addition to the preparation of high-quality film-like products, MOFs can be grown on the substrate in a certain direction by pre-functionalizing the substrate. In the study of Wang et al., they pre-functionalized a gold-coated silicon substrate with (111) orientation by depositing a self-assembled monolayer of 16-mercaptohexadecanoic acid (MHDA) and make HKUST-1 film along the (001) direction to grow on the substrate [59]. Park et al. also adopted the method of repeated wetting, which is that the Au bottom electrode covered polyethylene terephthalate (PET) was immersed in piranha solution to functionalize it with hydroxyl groups, and then obtain an active film with desired thickness through layer-by-layer deposition (Figure 13a) [71]. The synthesis of the product was proved by XRD and SEM characterization (Figure 13b,c).

**Figure 14:**
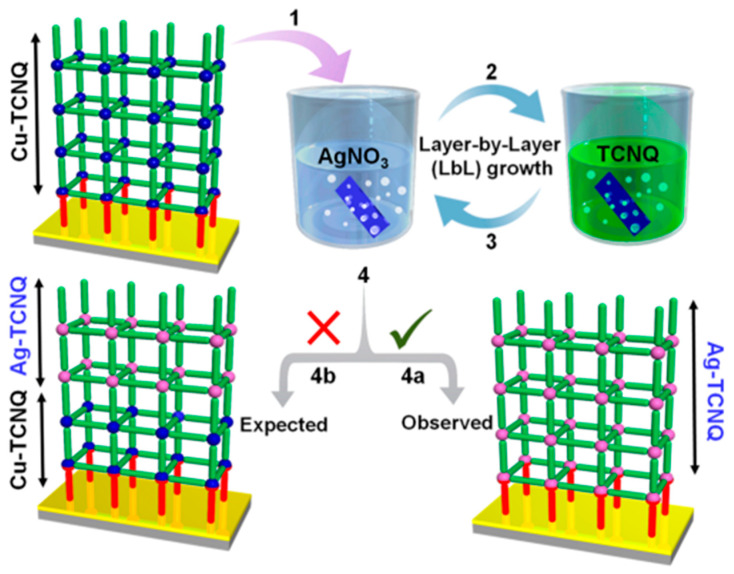
Schematic diagram of sacrificial LbL growth. Dipping of a preformed Cu-TCNQ thin film into AgNO_3_ solution (1) into TCNQ solution (2) and again into AgNO_3_ solution (3) Successive dipping into AgNO_3_ and TCNQ solutions makes one cycle of LbL. (4) All the original Cu-TCNQ films were sacrificed to obtain the (4a) structure with all metal elements replaced. Reprinted with permission [68] from ACS.

Some researchers also refer to it as the layer-by-layer synthesis method, which can synthesize the skeleton structure that is difficult to obtain by ordinary methods. Rana et al. replaced all copper in the pre-deposited Cu-TCNQ framework with Ag, thereby obtaining Ag-TCNQ with a Cu-TCNQ framework structure (Figure 14) [68], which is a structure that is usually difficult to obtain by solvothermal reactions. Although it appears to be a simple ion-exchange reaction on the surface, it is finally determined to be a redox reaction in which Ag^+^ oxidizes Cu^+^ to Cu^2+^ through the analysis of the growth of LBL films in each cycle by FESM and EDXS analyses.

#### 2.2.4. Template Method

The template method uses the original flat material as a template and allows the synthesized substances to be attached to the template in the shape of the material. The method is simple and practical to prepare material with the shape of the template, but a suitable template needs to be obtained first. The above-mentioned liquid phase epitaxy method is also a template method in essence.

**Figure 15:**
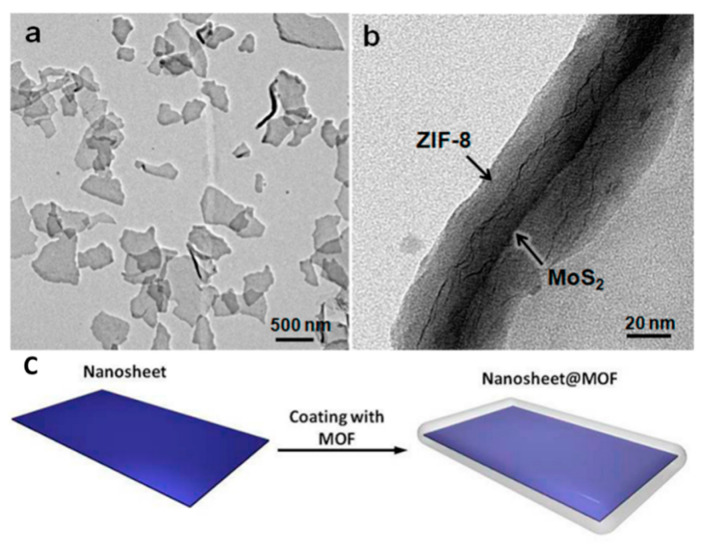
(**a**) TEM image of MoS_2_@ZIF-8 hybrid structures. (**b**) TEM image of a curled MoS_2_@ZIF-8 structure, showing the MoS_2_ nanosheet and ZIF-8 coating. (**c**) Schematic illustration of the formation process of nanosheet@MOF hybrid structures. Reprinted with permission [72] from ACS.

In the microstructure of MOFs, there are many layered 2D MOFs derived along the 2D plane direction, which allows this part of the material to rely on traditional 2D materials as templates to assemble molecules on the 2D plane. Huang et al. used the two-dimensional material MoS_2_ as a template and prepared a ZIF-8-coated MoS_2_ material by mixing the template with the ZIF-8 synthesis raw material reaction solution. The TEM characterization image and formation process are shown in Figure 15a–c [72].

#### 2.2.5. Microwave Heating

Through the frequent change of the polarity of the molecules in the material under the external alternating electromagnetic field, frictional heat is generated. Because the inside and outside of the material are heated uniformly and quickly at the same time, microwave heating method can increase the growth rate of the film to achieve rapid production. Precursors are usually dissolved or dispersed in solvents, and the mixture is then heated in a microwave oven for a few seconds to several hours to form the desired product [73,74].

Tran et al. synthesized another common MOFs material UiO-66 by microwave heating [60]. The reaction raw materials of zirconium tetrachloride (ZrCl_4_) and 1,4-phthalic acid (H_2_BDC) were simply dissolved in the mixed solvent of glacial acetic acid (AcOH) and N, N-dimethylformamide (DMF), and then the product was obtained after only 3 min in a microwave reactor. However, although this method is fast and has a high product quality, it generally only yields a powdery product.

#### 2.2.6. Interface Synthesis

Interface synthesis is a synthesis method that directly reacts on thin incompatible two-phase interfaces to obtain thin film products. The film can be directly transferred to the substrate by immersing the substrate in the reaction solution, which is a simple method to prepare large-area thin-film products without relying on the substrate.

**Figure 16:**
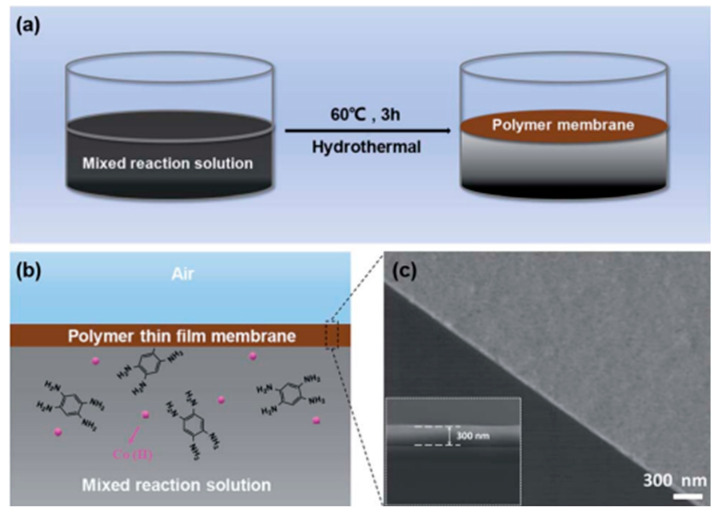
(**a**) Illustration of the polymer membrane formation process at the interface. (**b**) Schematic of the polymer membrane at the air-liquid interface. (**c**) SEM images of the as-prepared membrane with a thickness of 300 nm. Reprinted with permission [75] from RSC.

Zhang et al. fabricated large-scale d-p conjugated coordination polymer films at the gas-liquid interface through a mild coordination reaction between cobalt salts and ligands 1,2,4,5-phenylenetetramine tetrahydrochloride. The synthesis schematic diagram and preparation principle are shown in Figure 16a,b, and the SEM characterization diagram of Figure 16c, respectively [75]. The resulting brown membrane has a weak dependence on the substrate, making it can be easily transferred to any substrate by using a supporter to lift the membrane from the reaction solution. This characteristic makes it has a good practicability in various fields. According to the similar gas-liquid phase system, Liu et al. utilized the mild coordination reaction between cobalt salts and ligands 1, 2, 4, 5, 5-phenylenetetramine tetrahydrochloride (HHTP) to synthesize Cu_3_(HHTP)_2_ nanometer thin films at room temperature. The films have large transverse scale, high uniformity and thickness can be controlled by reaction time [76].

#### 2.2.7. Electrochemical Synthesis

Electrochemical synthesis of MOFs, as a minority method, has not been reported in the field of memristors. In view of its advantages that directly form films on the substrate or electrode and control their morphology and thickness, this method has a great prospect in the field of memristors, so we will briefly describe its characteristics.

As the name implies, this method is carried out by electrically driven synthesis. The electrode reaction can generate the metal nodes (metal ions or metal clusters), which are assembled with the active ligands in the electrolyte. Finally, the formed MOFs film will attach on the electrode surface [77].

In research of Cao et al., MOF-5 was electrochemically synthesized using a double zinc electrode and ammonium fluoride aqueous solution of BDC. In the electrode system, the anode zinc electrode is oxidized to release Zn^2+^ gradually, causing its concentration to greatly increases on the anode surface, yet the cathode electrolyzed water to produce OH^−^, which can react with the ligand BDC to deprotonate it. After these two reactions, a thin film of MOF-5 on the surface of the anode can be found [78].

**Figure 17:**
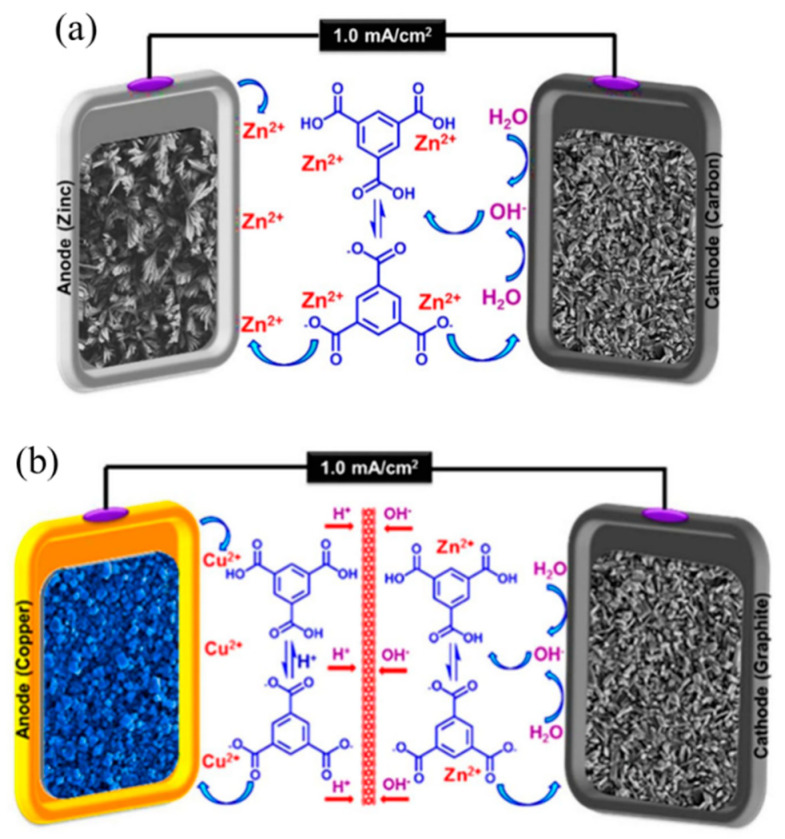
(**a**) Schematic diagram of the principle of simultaneous synthesis of Zn-MOF films with two electrodes. (**b**) Schematic diagram of the principle of simultaneous synthesis of Cu-MOF and Zn-MOF films with two electrodes. Reprinted with permission [79] from Springer Nature.

Alizadeh et al. reported two techniques for simultaneous growth of MOFs films on two electrodes at one time [79]. One is similar to the above example. The difference is that there is also an appropriate amount of free Zn^2+^ in the electrolyte. Because of the increase of OH^−^ concentration on the cathode surface, the free Zn^2+^ can also react with the activated ligand BTC to obtain the MOFs film on the cathode surface (Figure 17a). Another technology requires adding a separator in the middle of the electrolysis chamber in order to only pass H^+^ and OH^−^. Firstly, it is still dependent on the electrolysis of water to produce OH^−^ to activate the ligand in the electrolyte and crystallize on the cathode surface of the cathode chamber filled with the Zn^2+^ solution to obtain a Zn-based MOF film. Some OH^−^ passed through the middle partition to activate the ligands of the anode chamber. Because the anode chamber is not added by other metal ions, Cu-based MOFs films can be obtained on the anode surface after releasing the Cu^2+^ produced from the reaction (Figure 17b).

### 2.3. Device Structure and Electrical Parameters

**Figure 18:**
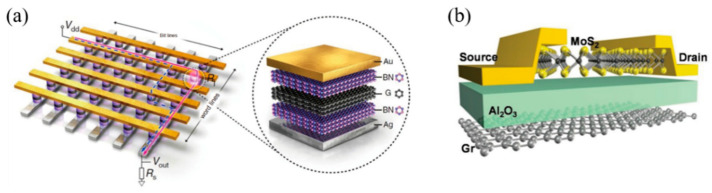
(**a**) Structure of two-terminal memristor. Reprinted with permission [80]. (**b**) Structure of three-terminal memristor. Reprinted from [81] with permission from Wiley.

The memristor is considered by the International Technology Roadmap for Semiconductors to be one of the most promising candidates for the next-generation in-memory computing architecture. Most memristors are two-terminal or three-terminal device structures, where the two-terminal memristor is an electrode/insulator/electrode sandwich (Figure 18a). Each layer of material is closely related to the overall switching performance of the device, and for most devices, the layers are also interdependent. The top electrode and the bottom electrode are usually used as the input port and output port of the signal respectively, and they are prepared with the same metal (such as Pt, Au, Al) to make the tested I–V curve have excellent symmetry. A three-terminal memristor is a structure that mimics CMOS (Figure 18b), and its three port functions correspond to the source, drain, and gate of CMOS. The non-volatile characteristic of three-terminal memristor enables it to realize the functions of traditional CMOS-based circuits and reduce circuit power consumption through the optimization of circuit structure. MOFs are composed of inorganic metals and organic ligands, which can form a huge extended library of MOFs by combination. Different materials of resistive switching layer will give memristors with different properties. By continuously optimizing the structure and performance of the MOFs, the MOFs-based memristor have better basic electrical properties than others. Next, we will introduce the basic device structure of the substrate, electrode, and functional layer, and the integration of the device. Finally, we will summarize the basic parameters of the classical memristor so that readers can compare the advantages of different devices.

**Figure 19:**
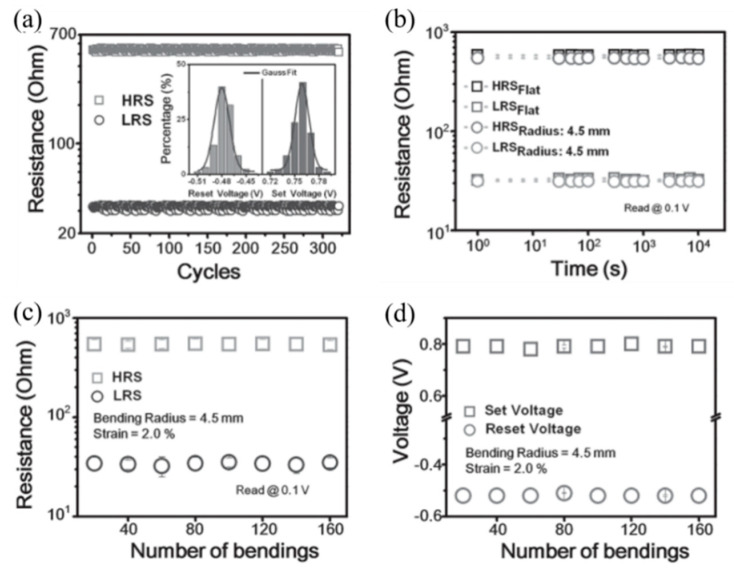
(**a**) Endurance performance of the Au/HKUST-1/Au/PET flexible device at the strain level of about 2.0% over 300 consecutive cycles. Inset of (**b**) Room-temperature retention performance of the high and LRSs of the flat and bended devices. Evolution of the (**c**) HRS/LRS resistances and (**d**) set/reset voltages of the Au/HKUST-1/Au/PET flexible devices as a function of the bending times at the strain level of 2.0%. Reprinted from [70] with permission from Wiley.

In order to make a bottom electrode with suitable thickness and high quality, it is also very important as a substrate for depositing the bottom electrode. Especially in flexible electronic devices, one of the important application fields of RRAM devices, the basic condition for making the devices bendable is to use a flexible substrate. In the work of Pan et al. [70], they used PET as the device substrate to fabricate a flexible RRAM that can withstand bending of 2.8% strain and maintain a uniform performance during repeated bending at 2.0% strain (Figure 19a–d), which is benefited from the inherent flexibility of the organic linkers in MOFs. Park et al. also used the substrate to increase the number of device bends to 100 times [71].

**Figure 20:**
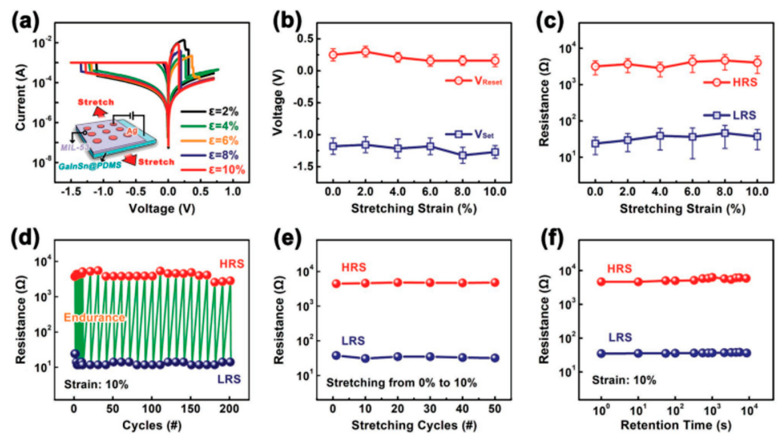
(**a**) Room-temperature current-voltage characteristics of the Ag/MIL-53/GaInSn@PDMS device under the stretching strains of 2–10%. Evolution of (**b**) the switching voltages and (**c**) HRS/LRS resistances of the Ag/MIL-53/GaInSn@PDMS device as a function of the strain level. (**d**) Endurance, (**e**) continuous stretching fatigue, and (**f**) retention performance of the device at the stretching strain level of 10%. Reprinted from [55] with permission from Wiley.

Beside organic flexible substrates, some liquid metals such as eutectic gallium-indium (EGaIn) and gallium-indium-tin (GaInSn) alloys are also important soft and elastic electrode substitutes in flexible electronic device [82,83]. In the work of Yi et al., they used the breathing material MIL-53 and the composite substrate of GaInSn and PDMS to increase the maximum strain to 10% [55]. At such large stretching strain, the memory device well maintains its non-formed bipolar resistive switching behavior, and under stretching at room temperature can hold for at least 10^4^ s (Figure 20a–f).

**Figure 21:**
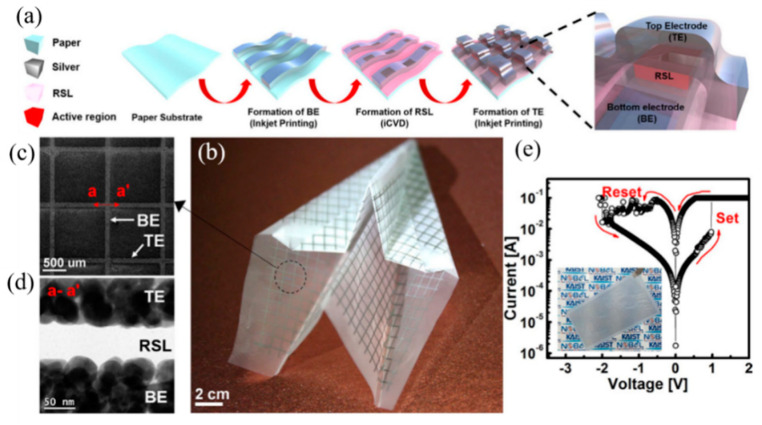
Fabrication process of paper substrate-based memory. (**a**) Schematics of the fabrication process. (**b**) Fabricated nanopaper-based memory. The memory is displayed in the form of an airplane prepared by folding, i.e., origami, demonstrating the foldable memory feature. (**c**) Top view SEM image of the fabricated device. (**d**) Cross-sectional TEM image of the direction along a–a′ of (**c**). (**e**) I–V characteristic of the fabricated device showing memory operation. Reprinted with permission [84] from Springer Nature.

Moreover, Lee et al. have perfected the process of paper substrates and successfully synthesized paper substrates using all-dry, solvent-free induced CVD techniques (Figure 21a) [84]. It is printable, disposable, foldable, and environmentally friendly (Figure 21b–e), as well as paper-based device can be completely destroyed to prevent hacking. It will surely have a place in the flexible substrate of RRAM devices in the future.

Electrodes as the basis of the device, it mainly plays the role of packaging and energization. There are extremely strict requirements for the thickness, quality, and material of electrodes. Currently, the thickness and quality of electrodes can be adjusted by regulating the process parameters of the electron beam deposition technology. Its common materials include Au, Ag, Cu, Si, ITO, FTO, and which electrode material to choose needs to consider the electrical properties and the influence of the material on the resistive switching effect.

**Figure 22:**
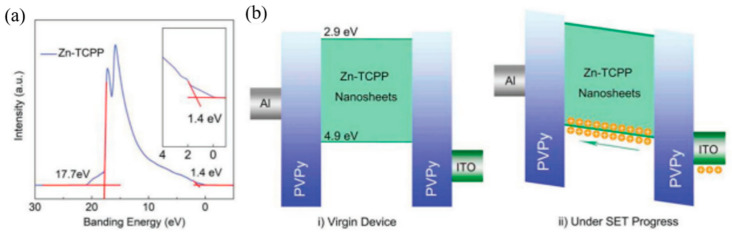
(**a**) The UPS image of Zn-TCPP nanosheets. (**b**) The energy band diagram of our Zn-TCPP nanosheet-based RRAMs. Reprinted from [53] with permission from Wiley.

The metal electrode also contributes significantly to the RRAM-type device mechanism in most cases. One of the more common functions is to form a Schottky barrier with the insulating layer/functional layers. The research of Ding et al. shows that the height and width of the Schottky barrier between electrodes and the insulating layer of Zn-TCPP@PVPy can be easily tuned by applying a bias voltage (Figure 22a) [53]. With the increase of the applied voltage, all trap sites in Zn-TCPP are filled, and the carriers can form conductive paths (CPs) to move freely, resulting in the resistive state from HRS to LRS (Figure 22b). The Ag-TCNQ produced by Rana et al. also established a Schottky barrier between the electrodes EGaIn, Ti or Pt, which shows a temperature-controlled change in electrical conductivity [68].

**Figure 23:**
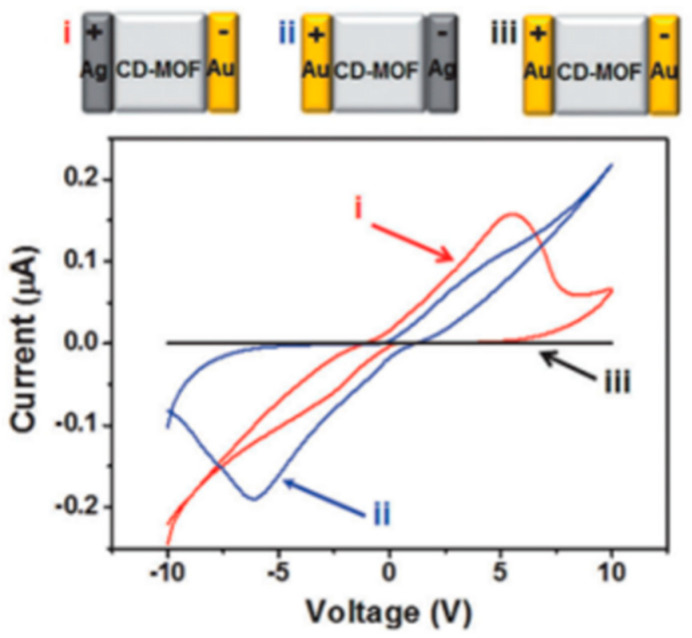
Cyclic voltammetry was performed on metal/MOF/metal structures in which the electrodes were made of (i) silver/gold, (ii) gold/silver, and (iii) gold/gold. Experiments were performed under ambient conditions with a scan rate of 0.07 Vs^−1^ and with 100 mm RbOH loaded into the MOF. The experiments reveal that memristor-type hysteresis and NDR require a positive (anodic) potential to be applied to silver, leading to its oxidation. Reprinted from [35] with permission from Wiley.

In addition, in some cases, the electrode material can also directly participate in the switching mechanism, which makes the electrode material more selective. In the report of Yoon et al., they found that both the top electrode (TE) and bottom electrode (BE) must use Ag material to achieve the resistive switching effect at both positive and negative potentials [35], because OH^−^ in Rb-CD-MOF can only undergo redox reactions on the surface of Ag (Figure 23). Experiments show that such a reaction does not occur on the surface of the Au electrode. Similarly, in some conductive filament mechanisms, conductive filaments are also formed by the migration of electrode materials after ionization, and reduction into conductive paths [60].

Insulator is the middle part of the device, also could be called as functional layer, which is the most critical part to realize resistive switching. The understanding of new high-electrical performance RRAM devices needs to start from this aspect. The design and mechanism of this layer also would be introduced in other chapters describe in detail.

There are some reports shows that several single crystal materials can be used directly to fabricate a complete device [59,67], but most bulk materials cannot have suitable resistive switching performance unless they are fabricated into nanometer-thick films [59]. Therefore, the film forming process of the functional layer is also an important step to improve the device performance. In addition to the methods of direct film formation during MOFs synthesis, such as template method [72], liquid phase epitaxy [55,56,58,69,71,72], and interfacial polymerization [75], the most classic film formation method for conventional materials is spin coating. The key to spin coating film formation is the viscosity of the functional layer system, so that some MOFs are doped by film-forming agents, such as PVPy (polyvinylpyrrolidone), PMMA (poly-(methyl methacrylate)) and PVA (polyvinyl alcohol) [54,60,66], to improve viscosity.

**Figure 24:**
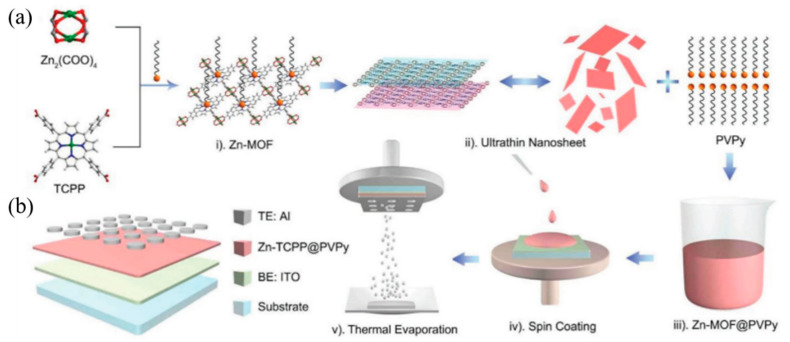
(**a**) The M-TCPP synthetic process. (**b**) The device preparation process of M-TCPP nanosheet-based RRAMs. Reprinted from [53] with permission from Wiley.

Spin coating is a film-making method that uses a solution to spread evenly on a rotating disc due to centrifugal force, with the advantages of simple process and adjustable thickness. As mentioned above, the M-TCPP material is mixed with the film-forming agent PVPy and then applied by spin [53]. In Figure 24, Zn-TCPP nanosheets mixed with PVPy are spin-coated on the glass substrate with ITO as BE. After annealing the film, 50 nm aluminum (Al) was thermal evaporated as top electrode (Figure 24a). Finally, the device with the structure of glass/ITO (185 nm)/M-TCPP@PVPy (35 nm)/Al (50 nm) is obtained (Figure 24b). Huang et al. and Tran et al. also spin-coated MOF materials on their respective substrate [60,73]. It is also worth mentioning that the follow-up studies show the film-forming agent PVA also has a certain influence on the conjugation effect of MOFs and switching mechanism.

So far, a number of MOFs-based memristors have been reported with electrical or optical properties [85], and its two-terminal devices can be ranged in the size from 20 μm to sub-100 nm to have many excellent properties, such as outstanding tolerance, wonderful retention, high on-off ratio, and low power dissipation. At the same time, it reported that the three-terminal memristor using a high-conductivity MOFs as field effect transistor is reported has the advantages of low power consumption and high conductivity. MOFs-based memristors have achieved milestone breakthroughs in single device fabrication and performance, but the in-memory computing architecture requires higher information density and faster signal transmission. As the number of devices increases, the array integrated by 2D planar will result an unacceptable signal delay. Moreover, it will lead to non-linear update of the weights, which will affect the learning accuracy and efficiency of the neural network [86,87]. The memory with 3D architecture, by contrast, can not only reduce the delay effect through shortening the connection distance, but also significantly reduce the interference caused by nonlinear weight changes during network training [88,89]. According to the port number of the memristor, the following section will be divided into 3D in-memory computing architecture of the two-terminal memristor and three-terminal memristor for discussion.

**Figure 25:**
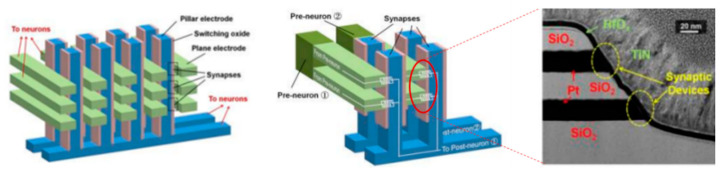
MOFs memristor-based 3D vertical neuromorphic circuit. Reproduced as open-access from [92].

In neuromorphic computing circuits, memristors have synaptic functions, and the stimulation and inhibition between the presynaptic and posterior membranes can be achieved by modulating ion transport in MOFs. The electrodes in the horizontal and vertical directions correspond to the two ends of the synapse, and the intersection is the resistive switching layer. The 3D in-memory computing architecture of the two-terminal memristor is shown in Figure 25, which is characterized by TEM. This 3D architecture can allow electrodes in the same direction to receive training pulses at the same time, thereby it has the dual advantages of improving the recognition accuracy of the neural network and reducing power consumption of device [90,91]. Considering this, a new architecture of gate transistor has been proposed that a four-layer vertical RRAM computing unit combined with CMOS [92]. This architecture can realize the logic of NAND (Not AND) gate and NOR (Not OR) gate by operating the specific pulse. It is worth mentioning that NAND gates are logically complete sets, which means that any circuit function can be implemented with NAND gates. Recently, an eight-layer memory-computing architecture array has been successfully fabricated [93].

**Figure 26:**
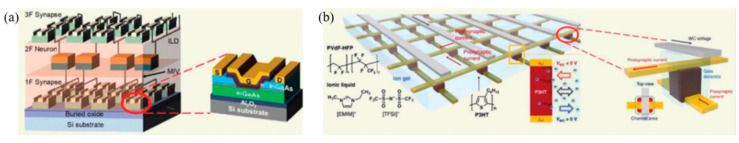
(**a**) 3D synapse-neuron integration process and single synaptic transistor structure. (**b**) CMOS-based 3D three-terminal memristor vertical in-memory computing architecture. (**a**) Reproduced with permission [96] from ACS. (**b**) Reproduced with permission [97] from Springer Nature.

Compared with two-terminal memristors, three-terminal memristors can achieve more complex input environments and higher controllability of synaptic weights [94]. In the biological brain, multiple synapses are interconnected, and three-terminal memristors can simulate its stimulation or inhibition to achieve multiple neural circuit transmissions [95]. To the three-terminal devices based on memristor, 3D memory-computing architecture is a necessary process for it to realize neuromorphic computing. Kim proposed a 3D integration method of synapses, neurons, and synapses by using traditional metal processes, as shown in Figure 26a. Similarly, three-terminal memristors can also be extended to 3D vertical memory computing architectures, as illustrated in Figure 26b. However, the integration of three-terminal devices is more difficult than that of two-terminal devices. Regardless of whether it is a two-terminal device or three-terminal device, there are always some problems in inorganic and organic memristors. With the emergence of MOFs, these problems are expected to solve by MOFs-based memristor.

**Figure 27:**
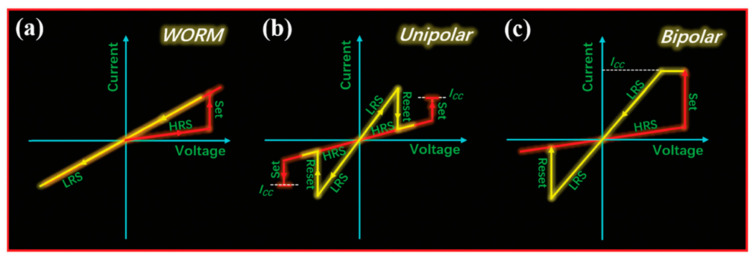
(**a**) WORM resistive switch, (**b**) reversible unipolar switch, and (**c**) bipolar switch. Reprinted with permission [98] from RSC.

In terms of device performance, we will also introduce the electrical parameters of the memristor. According to the characteristics of stored information, the electrical switching characteristics of RRAM devices can be divided into volatile and non-volatile types. According to the current-voltage (I–V) curves, nonvolatile switches can be generally classified into three types: write-once-read-many (WORM), unipolar, and bipolar hysteretic behavior. In WORM-type memory, the electrical switch from HRS to LRS is irreversible, i.e., the original state cannot be restored (Figure 27a). The unipolar and bipolar hysteresis behaviors both show electrically reversible switching behavior. The unipolar hysteresis shows the behavior of erasing and writing under the same polarity voltage (Figure 27b), but the bipolar memory devices need to operate at different polarities respectively (Figure 27c). Because RRAM has fast resistive switching and low power consumption, it is very advantageous in fast data writing, reading and erasing. Recently, the exploration of the application of RRAM in neural synapses or performing logical operations in memory has attracted extensive research attention to solve the Neumann bottleneck problem [98].

As an electronic component, the main indicators for measuring RRAM devices include switching ratio, retention time, cycle stability, erasing speed, operating voltage etc.

The switching ratio (I_ON_/I_OFF_) refers to the resistance ratio of high and low resistance states, which determines the degree to which a voltage is applied to distinguish the logic states of “0” and “1”. For current logic circuits, the power of 10 is more appropriate value.

The retention time refers to the effective holding time of the resistance state of the device, which is an important parameter for the non-volatile characteristics of the device.

The cycle stability refers to the effective erasing and writing times and quality of the device.

The erasing speed refers to the time required to switch between high and low resistance states. The shorter erasing time means more sensitive device.

The operating voltage includes threshold voltage (V_th_) and reset voltage (V_Reset_). V_th_ also is called set voltage (V_Set_) can make device change from HRS to LRS, which can be regarded as a writing process; the reset voltage is the voltage at which LRS changes back to HRS, which is an erasing process. The magnitude of two voltages determines the voltage operating range and power cost of device.

## 3. Memristive Switching of Metal Organic Frameworks

The designability of MOFs provides many conveniences for the design of RRAM devices, and research reports in recent years have also derived a relatively complete mechanism testing system. While there is no detailed and unified theory for many mechanisms, there are also many in-depth literature reports. In the following, we will discuss their effects on device performance from mechanism point to provide more ideas for molecular design.

### 3.1. Charge Trapping

**Figure 28:**
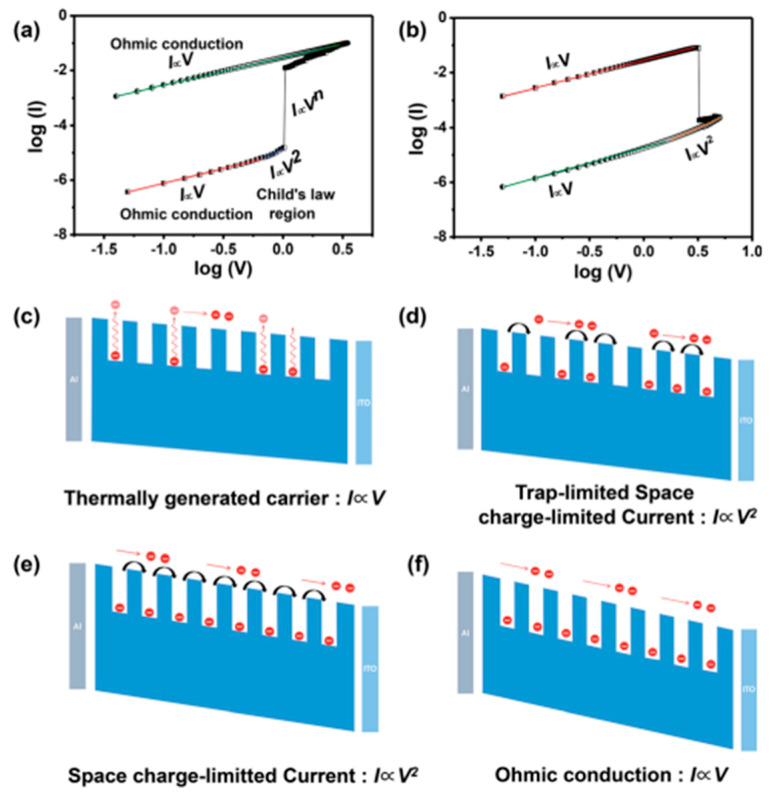
(**a**) Fitted I–V curves of the memory device at the set process. (**b**) Fitted I–V curves of the memory device at the reset process. (**c**–**f**) Transportation mechanisms of charge carriers from low conductive state to intermediate conductive state and further to high conductive state. Reprinted with permission [75] from RSC.

When the charge carrier density of the applied voltage injection is lower than the free carrier density produced by thermal excitation in the MOFs film, many traps or holes will be produced in the material to trap electrons [75]. At this time, the device shows ohmic conductivity. With the gradual increase of bias voltage, the space charge will appear, and the trap is gradually filled with the injected free carriers, resulting in a significant decrease in its density. At the same time, the formed built-in electric field in trap can shield the external electric field and further limit the carrier injection [67], so that the charge can move freely in the system to enable it with more excellent conductive properties (Figure 28c–f) [75]. This law is called trap limited-space charge limited conduction mechanism (TL-SCLC).

**Figure 29:**
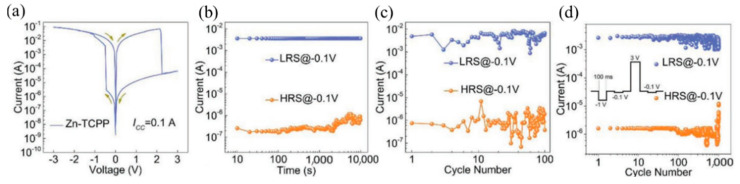
(**a**)Typical I–V curve of Zn-TCPP nanosheet-based RRAM device. (**b**) Retention test of Zn-TCPP nanosheet-based RRAM device. (**c**) Switching endurance of Zn-TCPP nanosheet-based RRAM device under I–V sweep mode and (**d**) under the pulsed voltage stresses mode. Reprinted from [53] with permission from Wiley.

Generally speaking, when the system is in the space charge limited conduction (SCLC) state, the charge movement is limited, and the device is HRS. When the trap is filled, the charge can move freely [53,66]. Obviously, the most direct way to verify this mechanism is to explore the I–V relationship in the operation of the device. At low voltage, the current is proportional to the voltage (I∝V, Ohm’s law), while it is proportional to the square of the voltage (I∝V^2^, Child’s law) as the voltage increases. (Figure 28a). According to this mechanism, the process should be reversible, i.e., the conduction law will return to the ohmic law, when the voltage decreases from high to low voltage (Figure 28b). However, the resistance value of the device changes to the LRS. It requires a higher negative voltage to reset the resistance of device to HRS, achieving the complete reversible process. In the M-TCPP-based device designed by Ding et al. [53], the Schottky barrier height and width formed between Zn-TCPP@PVPy and TE/BE can be readily adjusted by applied voltage bias. With the increase of applied voltage, all the trapping sites in Zn-TCPP have been filled and the charge carriers can form CPs to move freely, inducing to the RS from HRS to LRS. With the increased voltage bias from 0 to −3 V, the current will suddenly increase at −0.5 V (V_Set_ = −0.5 V), which indicates the device was switched from HRS to LRS. This process is named “programming”. With the voltage increasing from 0 to 3 V, a sudden decrease in current occurs at 2.5 V (V_Reset_ = 2.5 V), which is “erasing” process (Figure 29a).

There is almost no fluctuation after 10^4^ s retention test, 100 times sweep cycle, and 1000 times pulses cycle for Zn-TCPP nanosheet-based RRAM, which shows excellent memory stability and reliability (Figure 29b–d). By setting different compliance current (I_CC_) during the set process, the multistate reversible data level can be achieved.

**Figure 30:**
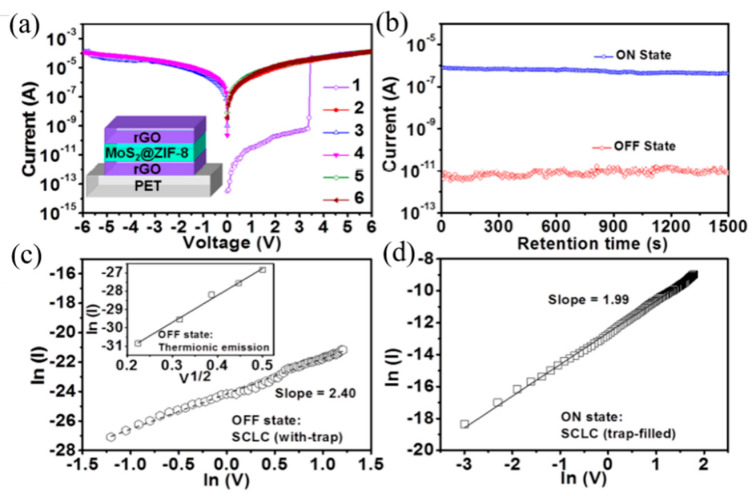
(**a**) The I–V characteristics of the MoS_2_@ZIF-8 based flexible memory device. Inset: the schematic of the device. (**b**) The retention-ability test of the memory device at a reading voltage of 0.5 V in the ON and OFF states. Experimental data and fitted lines of the I–V characteristics in (**c**) the OFF state and (**d**) the ON state. Reprinted with permission [72] from ACS.

The electrical test of the ZIF-8-coated MoS_2_ as the functional layer-based device developed by Huang et al. also showed SCLC characteristics [72]. Moreover, the trapped charges in MoS_2_ are retained after turning off the power, due to insulating ZIF-8 material is used as encapsulation matrix, which enables the high conductivity and nonvolatility of the memory device. The device also exhibits WORM memory effect: the device starts out in a low conductivity state (OFF state); the current state increases abruptly from 7.0 × 10^−10^ to 5.0 × 10^−5^ A (On state), when the applied voltage sweeps to 3.3 V; the ON/OFF ratio over 7.0 × 10^4^ at 0.5 V; it has yet to recover OFF state, even if the applied voltage is reversed to −6.0 V. It is the inerasable data storage characteristic (Figure 30a). The ON and OFF states of the device did not undergo significant fluctuation even after more than 1.5×10^3^ s of test under ambient conditions at a reading bias of +0.5 V (Figure 30b–d).

**Figure 31:**
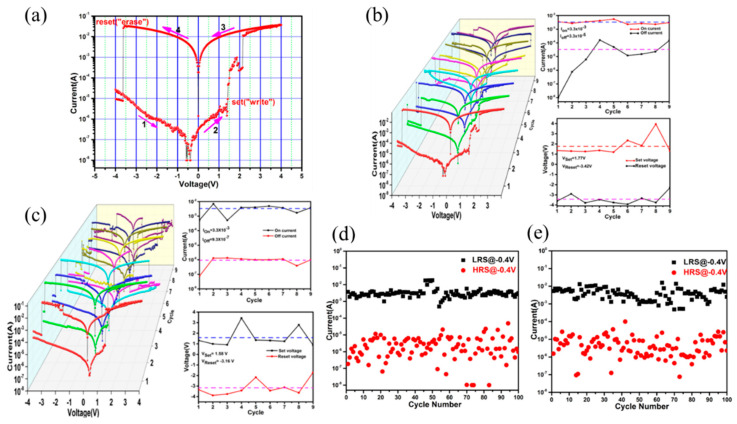
(**a**) I–V curve s of the ITO/POMOF/Ag device with one cycle; (**b**) I–V characteristics of the ITO/POMOF/Ag device at room temperature with nine cycles; (**c**) I–V curves treated by heating at 150 °C with nine cycles; (**d**) cycle endurance of the ITO/POMOF/Ag device at room temperature; (**e**) cycle endurance of the device treated by heating at 150 °C. Reprinted with permission [67] from ACS.

At present, there is no clear corresponding molecular structure for the trap, and the specific structure of the trap is also being explored. In the work of Chen and collaborators [67], they used XPS and UV-vis characterization and DFT calculations to demonstrate that under an applied voltage, the POM anion in the cationic framework of MOFs can accept electrons to realize the injection of electrons, resulting in a reversible reduction and the resistive switching behavior of the device (Figure 31a). The electron-poor metal viologen and molecular remodeling also contribute to the switching performance. The device they designed has an average on-off ratio of 100, can be cycled for 100 cycles, and is solvent/acid/base stable. The device can still show good stability without significant decrease in on-off current and cycle stability when heated to 150 °C (Figure 31b–e), and its bistable performance will reappear while cooling to room temperature.

### 3.2. Ion Penetration

MOFs materials benefit from their adjustable size, regular and rich pore structure characteristics, so that the transmission and penetration of ions inside the material have a smooth path. In the process of electrification, metal electrodes are often ionized, when the electrode ions can penetrate along the direction of the electric field is conducive to the generation of filamentous conductive mechanism. It is also known as electrochemical metallization (ECM) or conductive bridge (CB) mechanism, which is usually the resistance transition behavior in some regions of functional materials [99]. Since the filament size is much smaller than the device area, the formation has great randomness, but the greater the voltage, the stronger the directivity [53].

Among them, the metal conductive filament mechanism is reported in more literatures. The electrode metal is ionized under the action of an electric field, penetrates through the intermediate functional layer, and is re-reduced to metal with its help, leaving behind metal particles. Under the cumulative effect of the electric field, the metal particles accumulate to form a conductive filament that connects the positive and negative electrodes, so that the overall resistance of the device is greatly reduced. For the confirming this mechanism, the conductive filaments will be regulated by using different electrodes in control experiments [58,67], and then it is directly observed by the characterization methods such as AFM and XPS [56,60].

**Figure 32:**
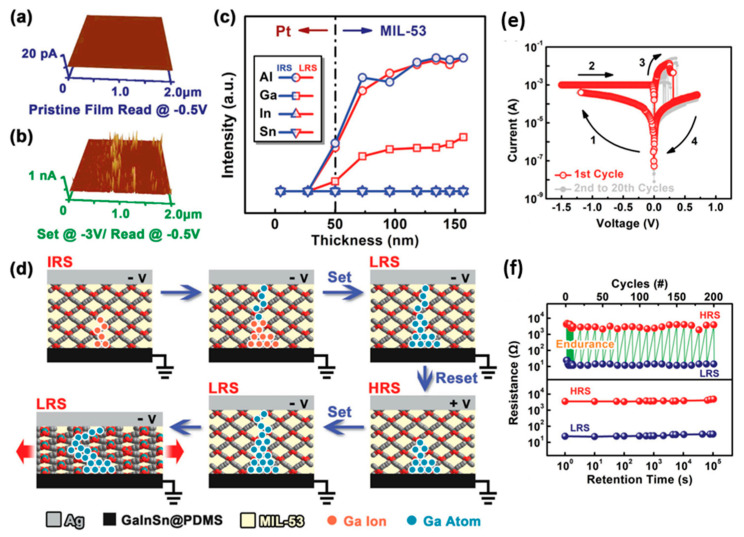
(**a**) Conductive atomic force microscopic current maps of the MIL-53/GaInSn@PDMS structure in its initial high state, and (**b**) after being set at −3 V. (**c**) XPS depth-profiling analyses of the Pt/MIL-53/GaInSn@PDMS device in its initial and low-resistance states. (**d**) Schematic illustration of switching mechanism in Ag/MIL-53/GaInSn@PDMS device. (**e**) Room-temperature current-voltage characteristics, and (**f**) retention and endurance performance of the unstretched Ag/MIL-53/GaInSn@PDMS device. Reprinted from [55] with permission from Wiley.

The Ag/MIL-53/GaInSn@PDMS device designed by Yi et al. was directly characterized by AFM and XPS data [55]. In the C-AFM measurement, several current response points with a diameter of ~10 nm were observed at a bias voltage of −3 V, which is in line with the characteristic of tiny diameter filamentous conduction paths (Figure 32a,b). When the device is switched to LRS, the electrode metal Ga and In signals appear at the same position, which indicates that the gallium in the galinstan alloy is injected from the soft electrode into the MOFs (Figure 32c). The XPS spectra shows that the Ga 2p_1/2_ and Ga 2p_3/2_ species are respectively at the binding energies of 1143.45 and 1116.69 eV in the LRS core-level. It confirms that the injected gallium has been electrochemically reduced to metallic Ga atoms, and they form an elastic conductive filament to connect the anode and cathode.

The phenomenological model of resistive switching illustrated in Figure 32d. When a negative voltage is applied to the top electrode, oxidation of the gallium atoms into the Ga^3+^ cations occur in the GaInSn@PDMS soft composite electrode. Under the applied electric field, the Ga^3+^ cations migrate across the MIL-53 layer and are reduced by electrons injected from the Ag electrode. When the opposite voltage is applied, the rupture/electrochemical dissolution of the Ga conduction path occurs at the weakest point, and the device reverts to HRS (Figure 32e). This device has forming-free bipolar switching characteristic with the ON/OFF ratio of ~200. Its HRS and LRS are programmable, accessible, and stable in the repeated switching cycles, and they can be readily retained for >10^5^ s (Figure 32f).

**Figure 33:**
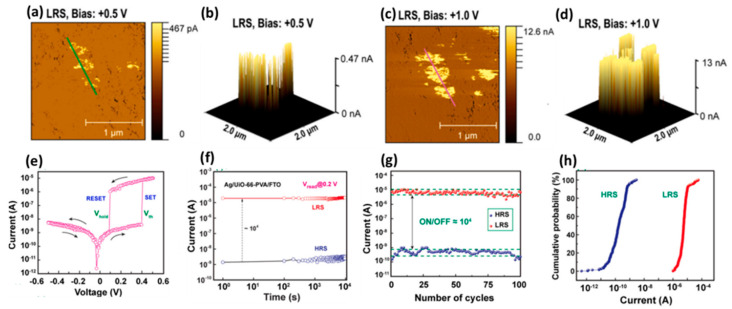
C-AFM images of UiO-66-PVA film drawn on silver substrate with bias voltages of (**a**) 0.5 V and (**c**) 1.0 V. Figure (**b**,**d**) is (**a**,**c**) three-dimensional C-AFM images, respectively. The Ag/UiO-66-PVA/FTO (**e**) in the voltage range of −0.5 → 0.5 V. (**f**)The retention time for data storage. (**g**) Endurance test during 100 cycles (**h**) and the cumulative probability during 500 cycles of Ag/UiO-66-PVA/FTO RRAM device. Reprinted from [60] with permission from Elsevier.

In the Ag/UiO-66@PVA/FTO device designed by Tran et al. [60], when a positive voltage was applied to the top electrode, the Ag electrode was oxidized and migrated between the functional layers of UiO-66-PVA, and some penetrated through the functional layer and was in the FTO. The bottom electrode is reduced to form nano-silver particles, which gradually form a small Ag conduction path. When the applied voltage is large enough, electrons can hop along the Zr_6_ junction or Ag^+^ in UiO-66. Such tiny filaments of conductive electrical signals were captured using C-AFM (Figure 33a–d). As a further verification, tests have shown that thicker conductive filaments are formed at higher voltages and hard to breakage, so the resistive switching effect is thus significantly reduced. At low voltage, the device has low operation voltage (V < 0.5 V), high ON/OFF ratio (~10^4^), excellent endurance (5 × 10^2^ cycles), longtime retention (10^4^ s). Its V_Set_ and V_Reset_ are 0.37 V and 0.07 V, respectively (Figure 33e–h).

The transport of ions is clearly not limited to metal ions. Using a redox mechanism similar to a primary battery, other ions can also be conductive in a smooth flow. Different from the direct migration of ionized electrode element ions, the components in MOFs can also directly participate in the redox reaction and produce ion transport charges. In the Rb-CD-MOF molecular memristor designed by Yoon et al., they made use of the redox mechanism that OH^−^ and silver electrode can be passivated to design a memristor with reversible characteristics [35]. The memristive performance varies with ion concentration and water content. The number of moved ions, as we know, determines the current conduction of MOFs. However, the dissociation energy of RbOH is very large under anhydrous conditions, so that there is a low ion content of Rb^+^ and OH^−^ in the system, and the material hardly shows conductivity.

**Figure 34:**
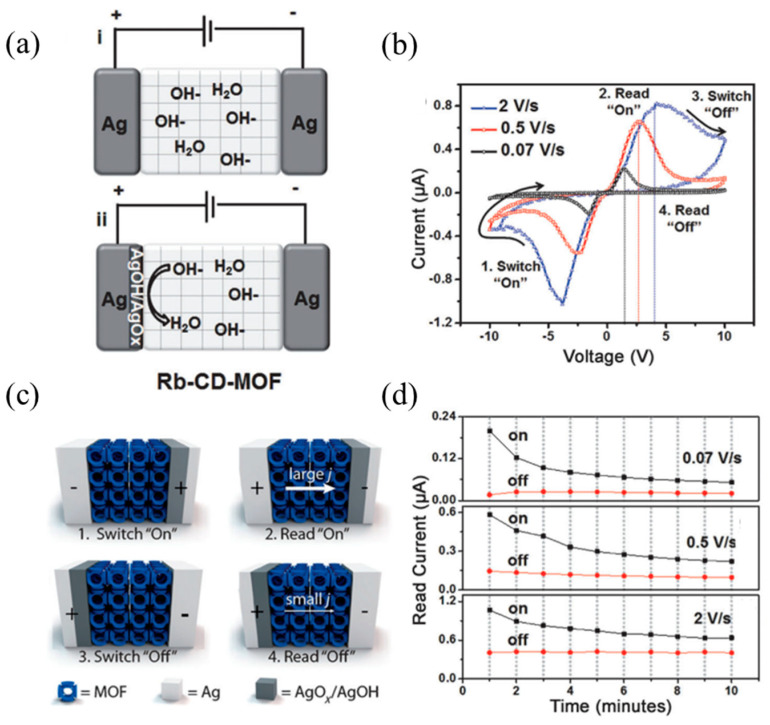
(**a**) A model of the electrochemical mechanism that leads to the switching behavior and memory effect in Ag/MOF/Ag devices. (**b**) Cyclic voltammetry is shown for three different scan speeds of a Rb-CD-MOF device flanked by silver electrodes. (**c**) In a full cycle, the system passes through two highly conductive states and two non-conductive states. (**d**) A “0” or “1” state was written into the MOF memory and repeatedly read with three scan rates. Reprinted from [35] with permission from Wiley.

Due to the limitation of the penetration of the electrode element Ag^+^ by the MOF sub-nanometer channel, the change of the apparent resistance of the device is generated by the redox process at the interface between OH^−^ and silver anode in MOF. Under alkaline conditions and positive potential, silver forms a layer of silver hydroxide (AgOH) and silver oxide (AgO_x_) on its surface (Figure 34a). This process is self-limited because further oxidation requires mass transport of hydroxide ions through the oxide layer. When the silver electrode is further oxidized and passivated accordingly, the initial current increases first and then decreases with the increase of voltage, which is the characteristic of complex differential resistance (NDR). The large Faraday current corresponds to the Read ‘On’ or ‘1’ state, and the reduction of current by passivating the electrode corresponds to the Read ‘Off’ or ‘0’ state. When the polarization is reversed, the original silver oxide layer and the other electrode are reduced and oxidized, respectively, so that the current reaches its maximum value again and then is weakens as the Faraday process completes (Figure 34b,c). In the cyclic voltammetry test, the initial negative energization process is regarded as a Switch ‘On’ state, and the complete process for the forward voltage is regarded as a Switch ‘Off’ state. It is worth noting that this memory is most clearly observed in wet nitrogen or air and disappears completely in dry nitrogen or dry oxygen. As shown in Figure 34d, the switching device has a distinguishable on/off ratio (~150) at the scanning rate of 0.07 Vs^−1^.

**Figure 35:**
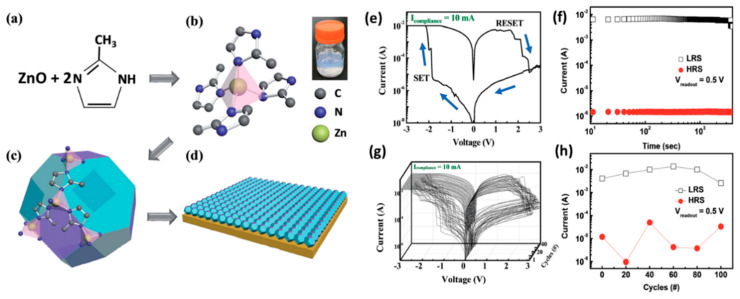
(**a**) Chemical formula with zinc and linker precursor. (**b**) One zinc atom and two 2-methylimidazole molecules combined to form tetrahedron nodes. (**c**) Metal nodes and imidazole linkers connected to construct a zeolitic structure. (**d**) ZIF-8 thin film coated on gold substrate. (**e**) I–V characteristics of the Au/ZIF-8/Al structured device. (**f**) Retention time analysis of the LRS and HRS states of the ZIF-8 ReRAM. (**g**) Resistive switching performance over repeated switching cycles. (**h**) Bending stability of the flexible ZIF-8 ReRAM with repetitive bending cycles. Reprinted with permission [71] from RSC.

There are also device studies that do not rely on electrodes to achieve redox performance. Park et al. achieved a redox memory mechanism by using an aromatic imidazole ring group ZIF-8 with redox properties [71]. The organic linker consists of an imidazole ring and a methyl group between two nonadjacent nitrogen atoms. When an electric field is applied on the device with Au/ZIF-8/Al structure, the zinc ions at the localized nodes act as hopping sites between organics (Figure 35a–d). At the set voltage, Zn^2+^ will be delocalized and reduced, and the remaining N atoms in the imidazole will be connected to form a conjugated conductive path, causing the system to become LRS. The conductive path will break when apply a positive voltage to the Au bottom electrode, and the system will change back to HRS. Two resistance state are stable during the holding test for 4000 s or cycle test for 40 times, and their on-off ratio is high to 10^4^. Meanwhile, the performance of device can be maintained over 100 bending test cycles (Figure 35e–h).

### 3.3. Skeleton Reorganization

In general, the MOFs skeleton is mainly used to support regular pores without the requirement of conductivity. However, due to the strong modification and regulation advantages of organic components, the materials can also be switched back and forth between high conductivity and weak conductivity through the conjugation of chemical bonds such as π bonds or the regulation of non-chemical bond forces such as hydrogen bonds, which coincides with the core electrical characteristics of RRAM devices. By switching the intramolecular configuration of MOFs, it is easy to regulate the formation of intramolecular π stacking and hydrogen bond [59,100], resulting in changes in molecular conductivity and a resistive switching effect.

**Figure 36:**
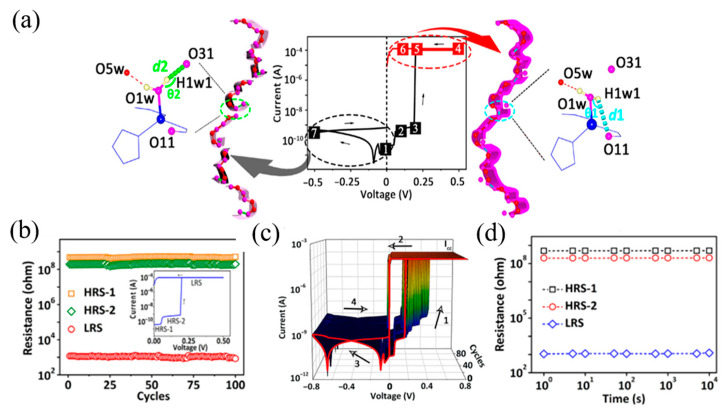
(**a**) The illustration of the applied DC voltages following the sequences as the points from 1 to 7. (**b**) Endurance performance (**c**) Semilogarithmic plot of the room-temperature I–V characteristics with 100 consecutive cycles. The red line represents the result of the first round of voltage sweeping. The arrows indicate the sweeping direction, while the numbers 1 to 4 represent the sweeping sequence. I_CC_ stands for the compliance current. (**d**) Retention performance of the three conductance states over 10^4^ s. The resistance of the sample was read at 0.05 V. Reprinted from [50] as open-access.

FJU-23-H_2_O is a kind of classical material which can adjust bistability by intramolecular hydrogen bond conversion [50]. In the absence of applied voltage, the electron density peak of H1w1 is toward O31 and deviates from O11. The hydrogen-bonded chain in the channel is divided into short-range hydrogen-bonded fragments by O1w hydrogen H1w1…O31 interaction, which make device in the HRS-1 state. When a DC voltage of 0.1 V is applied, two electron density peaks of H1w1 towards O11 and O31 are observed. This means that the hydrogen bonds of O1w H1w1…O31 and O1w H1w1…O11 exist at the same time. Therefore, the device is switched from HRS-1 to HRS-2. In Figure 36a, it shows the molecular structure before and after the transition from LRS to HRS-2. When the voltage increases to 0.5 V and then scans back to 0.2 and 0.1 V, the O1w atom is distorted and forms a hydrogen bond with O11. It will transform the short hydrogen bond fragments to interact with the complete hydrogen bond helix extending throughout the crystal, which keeps the resistance of device in the LRS when the bias voltage is lower than V_Set_. The rectifying effect occurs when a negative voltage is applied. The electron density peak of the H1W1 atom returns to O31 at a DC voltage of +0.5 V. The short-range hydrogen bond fragments were recycled again, resulting in a reset from LRS to HRS-1. From the test results in Figure 36b–d, the device has ultra-low set voltage (~0.2 V), long holdover performance (10^4^ s), high switch ratio (10^5^), and high rectification rate (10^5^).

The RSMOF-1 of Pan et al. can also undergo intramolecular hydrogen bond path transition under the action of an electric field [51]. Its resistive and ferroelectric switching properties are strongly regulated by the guest water molecules in the nanochannels, which utilizes the electric field-controlled hydrogen-bonding to interact with amino-linked RSMOF-1. The presence of amino groups in the channel provides abundant adsorption sites for water molecules through hydrogen bonding interactions. Changing the sample temperature will interfere the interaction with hydrogen-bonding and partially remove guest water molecules, which may significantly affect the ferroelectric properties of RSMOF-1.

First-principles MD simulations show that RSMOF-1 is nonpolar in nature, which means the formation of N·H-O·H-N bridge structures will occur in random orientations. Under the modulation of external electric field, the N·H-O·H-N bridge structure is forced to flip and arranges along the direction of the external electric field, resulting in an ordered-disordered ferroelectric polarization RSMOF-1. It will occur that the resistance transition from HRS to LRS when the majority of the bridging hydrogen bonds are aligned in the c direction.

**Figure 37:**
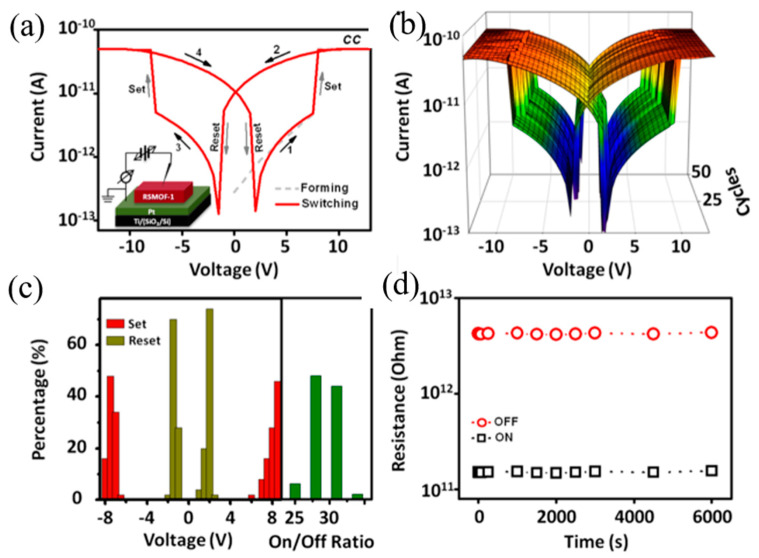
(**a**) Semilogarithmic plot of the room-temperature current-voltage (I–V) characteristics of the single crystal. (**b**) I–V curves and (**c**) distribution of the set/reset voltages and the ON/OFF ratio for 50 consecutive cycles. (**d**) Retention performance of the two conductance states over 6000 s. The resistance of the sample was read at 0.1 V. Reprinted from [51] with permission from ACS.

RSMOF-1 exhibits symmetric and bipolar resistive switching behavior at room temperature, as shown in Figure 37a of DC current-voltage (I–V) characteristics. When the voltage is swept in the direction of 0 V → 13 V → 0 V → −13 V → 0 V, the RSMOF-1 switches continuously between two resistive states, and this process has uniformly distributed threshold voltages (V_Set_ ≈ ±7.5 V, V_Reset_ ≈ ±1.5 V) and stable on/off ratio (~30) (Figure 37b,c). Tow states can be retained for at least 6000 s (Figure 37d).

In the modification of the chemical composition of the skeleton, some people have also tried to use the activity of the group to reorganize the skeleton structure. When Joule heating is performed, the linker with the carboxyl group and other heat-removable groups may lose the carboxyl group, and the remaining aromatic fragments will spontaneously connect to form a conjugated conductive molecular channel. Based on the in-depth analysis of XPS images, Pan et al. suggested that electric field-induced Cu^2+^ ion migration may be responsible for the uniform resistance switching observed in HKUST-1 nanofilms [70], which may lead to the subsequent pyrolysis of the trimesic acid linkers and the formation of highly conductive film.

**Figure 38:**
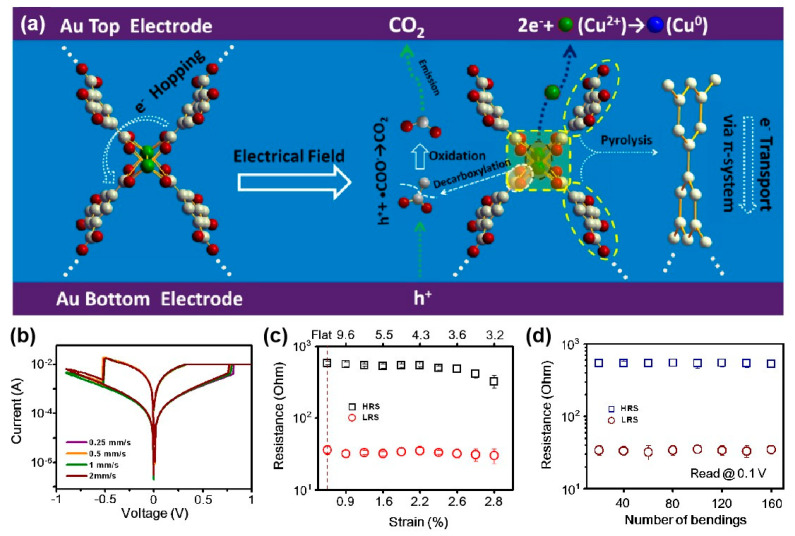
(**a**) Schematic illustration of the switching mechanism in HKUST-1 nanofilms. (**b**) Current-voltage characteristics during the dynamic bending test. (**c**) Resistance changes under different stress. (**d**) The maintenance of resistance value under multiple bending. Reprinted from [70] with permission from Wiley.

When a strong electric field is applied to the Au/HKUST-1/Au sandwiched structure, Cu^2+^ ions at local crystal defects can be transferred from the BTC linker to the gold layer and reduced to copper atoms. In HKUST-1 nanofilms, the negatively charged vacancies are relatively unstable, and heating can make carboxyl groups removes from the aromatic linker and releases as carbon dioxide through the top electrode. Then the pyrolysis of the triacid linker may lead to the coupling of adjacent benzene rings and the formation of sp^2^-hybridized carbon-rich channels. Carrier transport through this locally conjugated system will be more efficient than the transition between Cu^2+^ ions or BTC junctions, resulting in the device transitioning from a high-resistance state to an LRS. The reverse voltage can destroy the aromatic conductive filaments and reset the device to HRS, as illustrated in Figure 38a.

The device exhibits a uniform and repeatable resistive switching effect (V_Set_ = 0.76 ± 0.023 V, V_Reset_= −0.48 ± 0.017 V, on/off ratio = 18.5). Its high performance can be maintained in extreme application environments, the I–V curve is basically unchanged in the dynamic bending experiment of 0.25–2 mm/s, and it can withstand up to 2.8% tensile strain. The effective temperature of the device is between ±70 °C. No significant change in HRS/LRS resistance or set/reset voltage was observed when the device was bent for 160 times (Figure 38b–d).

### 3.4. Other Mechanisms

Different from the above more classical design ideas, there are also a small number of research reports on other properties of MOF materials. In view of the fact that there are few but novel studies in this field, here is still a brief summary of several unconventional mechanism forms.

**Figure 39:**
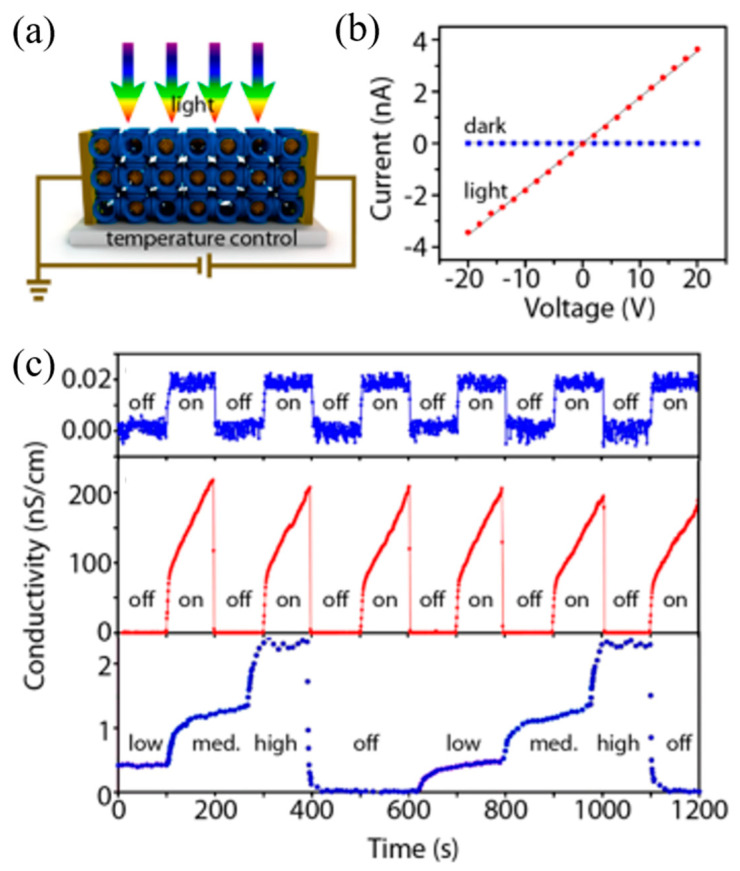
(**a**) Illustrates the experimental arrangement for measuring photoconductance of MOF crystals. (**b**) Two examples of I–V characteristics of AgNC@Rb-CD-MOF crystals under light irradiation (630 mW/cm^2^) and in the dark. The conductivities are, respectively. Changes in the conductivity of (**c**) a blank Rb-CD-MOF (first row), a AgNC@Rb-CD-MOF (second row) over several irradiation cycles. The bottom data are the recorded conductivity changes of AGNC@RB-CD-MOF when exposed to different power intensities of light under vacuum. Reprinted with permission [101] from ACS.

Tunneling is a form of transbond conduction, but the barrier between the donor part and the receiver part of the frame is too high to jump charge transfer. At present, only a small amount of literature has reported the related mechanism [30], which is controversial. Han et al. found that adding metal nanoclusters (NCs) to Rb-CD-MOF can make the modified materials have moderate electrical conductivity [101]. At the same time, its electrical conductivity increases by four orders of magnitude under light irradiation (Figure 39a,b), which results from the NCs distributing in the pores of MOFs. According to the report, the activation energy of thin film of metal NCs is consistent with the activation orbital model that the inverse cube root of the AgNC volume fractions (v^−1/3^) is proportional to the average distance between NCs, so the conductivity decreases exponentially with the increase of distance between NCs. In short, the electrical transmission in NCs-MOFs is realized through the tunnel transmission between NCs. As shown in Figure 39c, the characteristic of optoelectronic regulation makes MOFs crystal promising applications in optical switching device.

**Figure 40:**
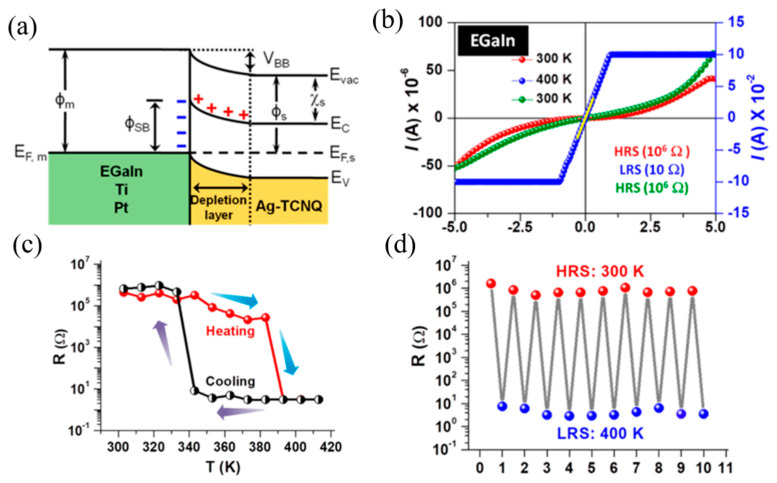
(**a**) Schematic of the energy diagram of the metal-semiconductor interface. (**b**) I–V characteristics of Ag-TCNQ thin films. (**c**) Heating and cooling curves of resistance versus temperature (R–T) clearly show a hysteresis loop vis-a-vis memory effect. (**d**) Plot of resistance values at HRS and LRS versus the number of cycles. Reprinted with permission [68] from ACS.

Since organic materials are generally afraid of high temperature, although the Schottky barrier principle indicates that the temperature-regulated memristor also has certain feasibility, the use of temperature regulation will still make the device risk of fire. Rana et al. synthesized Ag-TCNQ with Cu-TCNQ framework structure by LBL method [68] and formed the device with eutectic gallium-indium alloy EGaIn. After exclusion and verification, it was finally confirmed as a metal-semiconductor interface mechanism. Because the effective work function of Ag-TCNQ is much lower than that of EGaIn and the phenomenon of band bending, a Schottky-type barrier and depletion layer will appear at the interface (Figure 40a). The electronic property of device exhibits non-ohmic conduction and ohmic conduction at room temperature and high temperature respectively, which is a characteristic of temperature-controlled switching characteristic. The device realizes the reversible switching from HRS to LRS between 300 K and 400 K, and its switch ratio is close to 10^5^. The distinct characteristics of thermally driven reversible resistance switching (HRS↔LRS) are shown in Figure 40b–d.

## 4. Application of MOFs-Based Memristor

Limiting to the traditional architecture of von Neumann and CMOS technology, there is a balance between the device count, operating frequency, and circuit performance. Each time the CPU processes data, it needs to re-read the data in the memory, resulting in increase of power consumption and latency. The binary information storage capacity has severely limited the CPU’s energy-efficient data processing speed. At the same time, the device has only two states of on and off, which requires complex circuit structure when it calculates high-order tasks. In the post-Moore era, developing devices with new computing architectures are important to continue Moore’s Law and break through the performance of chips. With the in-depth research into biological brains and the rapid development of memristors, a state-of-the-art architecture of in-memory computing has been proposed and attracts with widespread attention. Neural network algorithms and device 3D integration lay the foundation for neuromorphic hardware circuits. Extremely high work efficiency and power have stimulated the potential of edge computing, enabling more artificial intelligence devices in life.

### 4.1. Data Storages

**Figure 41:**
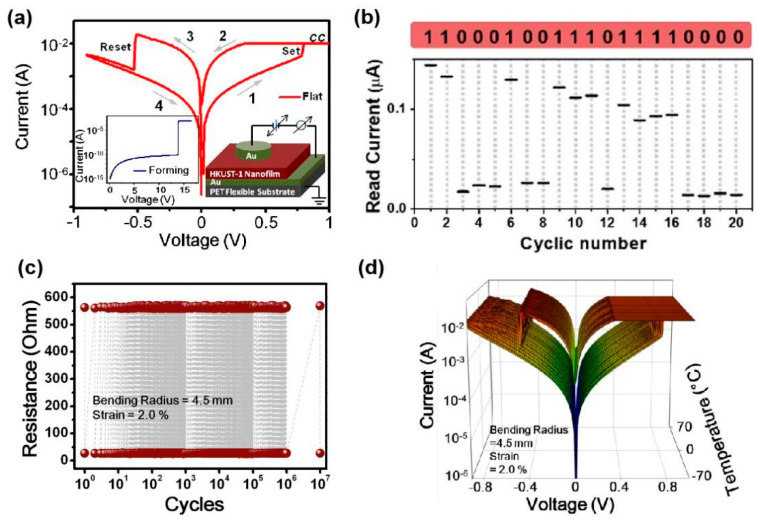
(**a**) I–V curves of Au/HKUST-1/Au/PET memristor [70]. (**b**) Performance of the memristor-based memory. Reprinted from [35] with permission from Wiley. (**c**) Resistive state retention. (**d**) Temperature disturbance and cycling tolerance of the device. (**a**,**c**,**d**) reprinted from [70] with permission from Wiley.

The electrical properties and non-volatility of MOFs-based memristors have excellent potential in resistive memory. The memristor changes its resistance under the action of voltage or charge, and this change will remain when the external stimulus is removed. The highest resistance value and lowest value are defined as logic ‘0’ and ‘1’of binary information storage, respectively. Compared with traditional CMOS devices, memristors theoretically have infinite resistance states and have excellent information density. The voltage required for a memristor to change from HRS to LRS (Set process) is the threshold voltage of V_Set_, and there is a threshold voltage of V_Reset_ for LRS to HRS (Reset process). The level of the threshold voltage is directly related to the switching speed of the resistance state and the power consumption. The research time of MOFs-based memristor is relatively short, but it is believed that the excellent performance of MOFs will soon revolutionize the commercialization of existing memristor, which led by inorganic materials and organic materials. In 2014, Yoon selected Ag as the terminal electrode and Rb-CD-MOF single crystal as the resistive switching layer to prepare the first MOFs-based memristor, its hysteresis curve has proven it is a memristor, even if the resistive switching needs ultra-high threshold voltages. The concept of a MOFs-based memristor as a non-volatile memory is validated (Figure 41b). Memory performance is mainly related to storage capacity, access time, bandwidth, and period, therefor community researchers are driven to optimize the basic electrical properties of MOFs-based memristors, such as area, threshold voltage, switching speed, tolerance, and retention. In 2015, Pan developed a new MOFs material of HKUST-1 for memristor, and its I–V curve shows excellent symmetry and low threshold voltage, which means the device has a low power consumption (Figure 41a). The device can achieve more than 1.0 ×10^7^ c ycles (Figure 41c), and still has outstanding resistive switching effect under the extreme temperature disturbance of −70–70 °C (Figure 41d). However, its small on/off ratio makes device hard to distinguish the resistance state during high-frequency reading and writing. Park proposed an Al/ZIF-8/Au/memristor with excellent on/off ratio (10^6^) [71], low power dissipation (V_set_ = 1~2 V) and retention (4 × 10^3^), but the reported number of cycles is only 40. Furtherly, a MOFs-based memristor with Ag/PVA-UiO-66/FTO/glass structure was reported [60], which has reasonable endurance (500), retention (10^4^), threshold voltage (0.5 V), on/off ratio (10^4^), and size (area of modification is 50 nm). With the continuous emergence of new MOFs with high-performance, MOFs-based memristors will appear to be more suitable for non-volatile memory.

### 4.2. Artificial Synaptics for Neuromorphic Computing

In the biological brain, information transmission between neurons is achieved through synapses. By imitating the mechanistic principles of biological brain computing, the synthesized MOFs-based memristor will realize the synaptic plasticity of the device. In the neuromorphic circuit that the device simulates the synapse, the top electrode and the bottom electrode correspond to the presynaptic membrane and the postsynaptic membrane respectively, and the resistance value represents the weight value of the neural network. According to the types of signals transmitted by synapses, they are divided into chemical synapses (generally mammals) and electrical synapses (common in fish and amphibians). MOFs-based memristors also have electrical and optical synaptic devices corresponding to external stimuli.

#### 4.2.1. Electrical Artificial Synaptic

**Figure 42:**
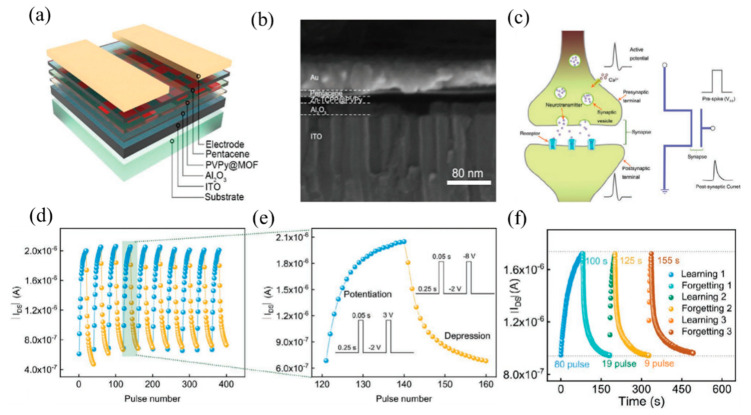
(**a**) Three-terminal memristor structure based on Zn-TCPP. (**b**) The SEM image of synaptic. (**c**) Circuit schematic of the synapse. (**d**) Current variation of the device upon repeated application of positive and negative pulses. (**e**) Current variation of the device during one cycle (amplitude: 3 V and −8 V, width: 0.05 s, interval: 0.25 s, base voltage: −1 V, VDS = −5 V). (**f**) Learning-forgetting curve of the MOF memristor-based device (amplitude: 5 V, pulse width: 0.5 s, interval: 0.5 s, base voltage: −1 V, VDS = −5 V). Reprinted from [102] with permission from Wiley.

**Figure 43:**
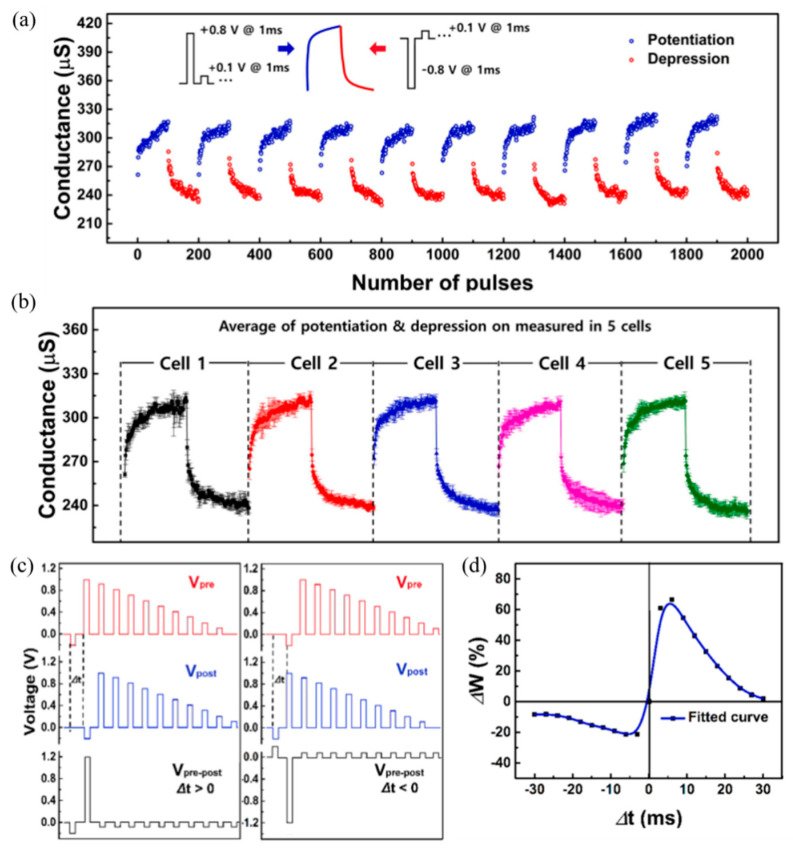
(**a**) Current variation of the device when positive and negative pulses are repeatedly applied. (**b**) Average of five values for the potentiation and the depression results measured in five cells. (**c**) Schematic diagram of pulses applied to test STDP properties. (**d**) STDP characteristics of the device. Reproduced from [103] with permission from Elsevier.

Ding and colleagues proposed the first MOFs-based memristor to achieve synaptic plasticity with both learning and signaling functions. They proposed the synthesis process of the Zn-TCPP MOF and the structure of synaptic based on it are shown in Figure 42a, and Figure 42b, respectively. The corresponding relationship between biological synapses and hardware circuits is vividly displayed (Figure 42c). Changes in the current of the device over 10 cycles (Figure 42d) and a single cycle (Figure 42e) were recorded. It was found that when 20 consecutive positive pulses of 3 V were applied, the device current rapidly rose to 2 μA, and then it returned to slightly larger than the initial value while applying 20 consecutive negative pulses of −8 V. The experimental results show that the synapse based on Zn-TCPP MOF has excellent conductance change and consistency, which can be well used to simulate the change of synaptic weight value (potentiation and depression) under the action of signal. The learning and forgetting processes of synaptic devices are verified to be biologically consistent, as shown in Figure 42f. The number of pulses required for the first, second, and last learning are respectively 80, 19, and 9. On the contrary, their forgetting times gradually increase and in order as follows: 100 s, 125 s, and 155 s. While the device demonstrates multiple synaptic plasticity, it requires the application of pulses of very high amplitude and low frequency, which is disadvantageous in high-speed neuromorphic computing. Later, Jeon proposed a high reliability synaptic device (Ag/ZIF-8@PVP/ITO). The device has excellent electrical properties, such as operating voltages of 1.24 V and −2.75 V, and high on/off ratio (7.8 × 10^3^). With 100 pulses as a cycle, i.e., 50 potentiation and 50 depression, the conductance value can be stably regulated within 10 cycles (Figure 43a). The absolute value of enhancement and suppression voltages are same of 0.8 V, and the pulse frequency reaches the level of 1.0 MHz (1.0 × 10^6^). It is evaluated that the conductance value changes of five cells during the enhancement and inhibition process (Figure 43b), which shows that the device has good consistency and symmetrical regulation process. Although the device can control the conductance value stably and reliably within 10 cycles, the conductance begins to drift in the 11th cycle. It is worth mentioning that the device has the Spike Timing Dependent Plasticity (STDP) feature related to timing, which is a necessary feature to realize the new generation of Spiking Neural Network (SNN). The effect of the pulse direction on the synapse and the effect of the arrival time of the presynaptic and post-synaptic pulses on the synapse need to be considered. The definition of Δt is shown in the Figure 43c, and STDP is similar to a typical timing asymmetric form Figure 43d.

#### 4.2.2. Optical Artificial Synaptic

**Figure 44:**
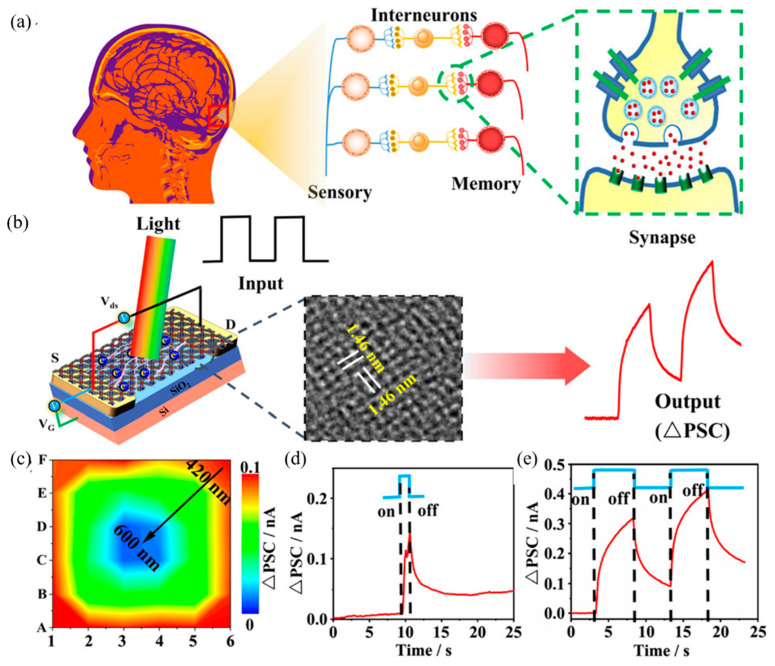
(**a**) Information processing architecture in the human brain. (**b**) 2D MOF-based synaptic device structure. (**c**) Current changes of the MOF synaptic array under different wavelengths of irradiated light. (**d**) Current changes of MOF synapses under 420 nm wavelength light pulse (V_ds_ = 1 V and V_G_ = 0 V). (**e**) Changes in synaptic currents under continuous stimulation with 420 nm wavelength light pulses. Reprinted from [104] with permission from Wiley.

Liu and colleagues first reported a novel 2D MOF material (Cu-THPP), and the device based on it exhibits excellent synaptic plasticity under light stimulation. The realization of opto-synaptic devices is based on the response of the MOF molecular structure to light stimuli. The optical device still maintains good stability and high response in air, and it has great potential for future robotic retina applications. As shown in Figure 44a, the conductance state of synapse is adjusted after sensing the light stimulation, and then it transmits the information to the next neuron. An optical signal was applied at the gate of the 2D Cu-THPP-based synaptic device (Figure 44b), and its performance was evaluated by testing the current changes in the source and drain, which respectively correspond the membranes of presynaptic and post-synaptic. Through applying different wavelengths of light stimulation to the synaptic array, the results show that the MOFs material is most sensitive to 420 nm wavelength and has a best current response (Figure 44c). The current of MOFs-based synaptic devices rapidly generates or gradually decreases as the light is turned on or off, which is similar to the short-term potentiation (STP) characteristic (Figure 44d). Through applying multiple identical light pulses in succession, the obtained current curve resembles the genetic memory curve in biology, as shown in Figure 44e. In addition, the synaptic device was positively correlated with light time and light intensity, and the gate voltage amplitude was inversely proportional to the device response time. The results of the study are in line with the laws of human learning and memory. For example, the former reflects that human brain learning is proportional to time and intensity, and the latter reflects individual differences in learning between different people.

### 4.3. In-Memory Computing Chips

**Figure 45:**
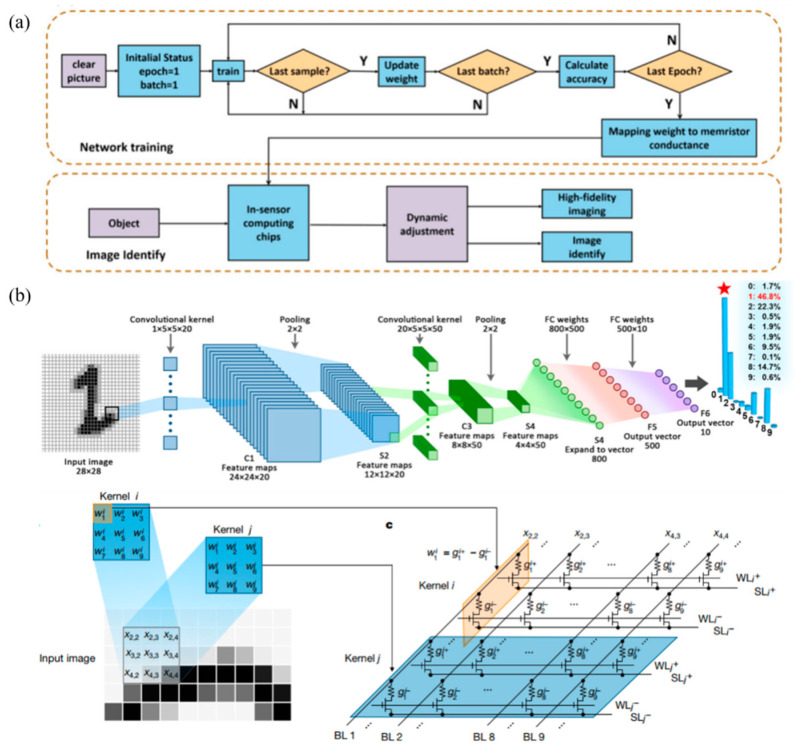
(**a**) Memristor-based convolutional neural network algorithm. Reproduced with permission [106] from MDPI. (**b**) Hardware in-memory compuing circuit architecture for digit recognition. Reproduced with permission [16,107] from Springer Nature.

Brain-inspired computing is based on a large amount of data for network learning and training, which places huge demands on memory and processors. To the traditional computing architectures, it often takes several weeks or even months. The non-volatile nature of memristors allows the device to fuse memory and computation in a single unit, resulting in a significant time and energy saving, which is the basis for enabling in-memory logic operations and neuromorphic computing. As the active layer of memristor, MOFs films have shown great application prospects in electrical (optical) synapses and in-memory logic operations. Although MOFs-based memristors are less explored in the field of neural networks, it has been verified that the hardware circuits based on other memristors are used to pattern recognition [27,105], especially for digit recognition based on convolutional neural networks. Figure 45a shows the algorithm flow chart of Fashion-MNIST (Mixed National Institute of Standards and Technology database) recognition based on memristor. During the neural network training process, the network weights are iteratively updated according to the error calculated by the algorithm. The synaptic weight is the actual device conductance value. The signal input and result output of system are the voltage pulse and the current, respectively. Therefore, the conductance value and the number of pulses is required to have excellent linearity, and it is easier to calculate that the number of pulses required to update the weights, which improves the training efficiency and reduced the power consumption. Finally, input a large number of pictures to test network performance, such as recognition accuracy and loss function. The hardware circuit architecture and Convolutional Neural Networks (CNN) algorithm flowchart are shown in Figure 45b. The memristor array implements the matrix operations involved in the pooling and convolution process, and the fully connected layer correlates the local characteristics in the hidden layer to judge the output result by analyzing the current value of each neuron.

## 5. Conclusions and Outlook

Over the past two decades, there have been tremendous advances in memristor and material of MOFs. Memristors have the advantages of scalability, in-memory logic computing, and compatibility with CMOS, leading to systematical researches for them in circuit design, neuromorphic computing, and device design. Abundant organic ligands and metal ions can create a large number of MOFs with specific structural and functional properties. Hence, the Memristor based on MOFs have the advantages of fast flipping, size scalability, high on-off ratio, and low power consumption, and degradability. In this review, we systematically summarize and discuss the progress of MOFs-based memristor from aspects including fabrication process, resistive switching mechanism, and potential applications in storage and neuromorphic computing.

At the material level, constructing high-density and uniform thin films and controlling the electrical properties of MOFs by tuning the metal ions or guest species adsorbed in the pore space remain great challenges. It is worth mentioning that recent studies have shown that high electrical conductivity and mobility can be achieved by changing the structure of MOFs, such as two-dimensional conjugated MOFs and MOF skeletons with permanent pores, and the use of bimetallic MOFs improves the conductivity of thin films [108,109]. In addition, the compatibility of MOFs with other materials highlighted in the roadmap is still an urgent problem to be solved in the application of MOF memristors. Although the hot solvent method has excellent versatility, it is difficult to control the thickness and orientation of film by spin coating, which often needs the support of film-forming agents. This problem can be solved by film-forming synthesis methods, such as liquid phase epitaxy and interface synthesis. Importantly, the electrochemical synthesis can also well realize the adjustable morphology and thickness of the film. However, the above film-forming synthesis methods often have complicated steps or harsh conditions, which means the selection of synthesis method needs further trade-offs in the actual application scenarios.

At the device level, it is necessary to deeply understand the resistance-switching mechanism and device manufacturing technology to give full play to the device performance in practical applications. For neuromorphic and memory computing, logical operations can be performed by applying optical signals and electricity on the device, and synaptic plasticity can be achieved by tuning metal ions of device. At present, the material design and theoretical calculations of the device are still in the exploratory stage. It should be clarified for the resistive switching mechanism of the resistive switching layer material to improve the devices performance.

At the application level, MOFs have a unique combination of properties that enable applications that cannot be achieved with traditional organic or inorganic materials alone. In the future, MOFs-based memristors will develop towards more dimensions and higher density integration. We also believe it will guide the industry to realize the off-site integration of the three functional modules of sensing, storage, and computing, ultimately removing the physical barriers and breaking the architecture of von Neumann.

In addition, although MOF memristors are currently in the stage of simple performance testing, they still have great potential to replace CMOS devices and make breakthroughs in green devices, neural computing, and information security. For green recyclable devices, although existing MOFs are classified as green materials and play a key role in low-carbon environmental protection, there is still a need to create more degradable and recyclable MOFs. For neuromorphic computing, it is necessary to further optimize the device performance to achieve more synaptic plasticity, such as LTP, LTD, STDP, SRDP, etc., as well as the design of neural network architecture for the device adapters. To further promote the development of IoT information security, it is necessary to design hardware security primitive circuits by utilizing the inherent characteristics of devices.

Summarily, the potential of memristors exhibited by MOFs is of great scientific significance in the post-Moore era. As the roadmap is continuously updated, the community focuses on key core issues and jointly promotes the development of MOFs-based memristors. It is believed that there will be more applications of MOFs-based memristors in the next decade, and devices may also develop from laboratory devices to industrial devices.

## Figures and Tables

**Table 1:** Performance list of Memristors based on MOFs or some other materials.

Device Structure	Memory Effect	SET/RESET Voltage [V]	ON/OFF Ratio	Retention [s]	Endurance	Ref.
Ag/Rb-CD-MOF/Ag	unipolar ***	±2~4/±10	10	>10^5^	~20	[35]
Ti/TiN/Cu_x_O/Cu	bipolar	2.8/1.1	10^3^	>10^5^	6 × 10	[38]
ITO/EVA:ZnO NPs/ITO	WORM	4	10^3^	4 × 10^4^	-	[39]
ITO/PMMA/CsPbBr_3_/PMMA/Ag	bipolar	2.6/−2.8	6×10^5^	>10^4^	5000	[40]
Ag/h-BN/Cu foil	bipolar	0.72/−0.37	1 × 10^2^	3 × 10^3^	550	[41]
Ag/MoS_2_@PVA/Ag	bipoalr	3/−3	1.28 × 10^2^	1 × 10^5^	1 × 10^3^	[42]
Mg/Ag-doped chitosan/Mg	bipolar	1.63/−0.82	>10^2^	>10^4^	>60	[43]
ITO/1 */Ag	bipolar	0.52/−0.4 (270 °C)	55.5 (270 °C)	-	-	[44]
Al/PEDOT: PSS-EB-NCNT/Al	bipolar	−2/3	10^3^	1 × 10^4^	100	[45]
ITO/Ni-BPTA/Al	unipolar	−1.65/−2.81	1:10^4^:10^8^ *	>10^4^	10^4^	[46]
Ag/ZIF-8/Au/PDMS	bipolar	3.5/−1.4	-	3000	10	[47]
Ag/UiO-66@PVA/FTO	unipolar	0.37/0.07	~10^4^	10^4^	5 × 10^2^	[48]
Ag/FJU-23-H_2_O/Ag **	bipolar	0.2/−0.5	10^5^	10^4^	100	[49]
Ti/(SiO_2_/Si)/Pt/RSMOF-1	bipolar	±7.5/∓1.5	∼30	>6000	50	[50]
ITO/POMOF/Ag	bipolar	1.77/−3.42	10^2^	-	100	[51]
Al/Zn-TCPP nanosheets @PVPy/ITO	bipolar	−0.5/2.4	10^3^	10^4^	10^3^	[52]
Au/HKUST-1/Au/PET	bipolar	0.76/−0.48	18.5	10^4^	10^7^	[53]
Ag/MIL-53/GaInSn@PDMS	bipolar	−1.2/0.3	200	>10^5^	200	[54]
(PET/Ti/)Au/ZIF-8/Al	bipolar	−1.9/~1.5	~10^4^	4000	-	[55]
(PET)/rGO/MoS_2_@ZIF-8/rGO	WORM	-	7.0 × 10^4^	1500	-	[56]

1 * = [Co(H_2_O)_6_]_2_[Co_3_(bpdo)_4_(H_2_O)_10_][Co_4_(H_2_O)_2_(B-a-PW_9_O_34_)_2_]·2bpdo·14H_2_O. * The device has three resistance states. ** MOF single crystal test conditions. *** The basic concepts of the parameters can be found in the introduction to Section 2.3.

## Data Availability

Not applicable.

## Abbreviations

IC	Integrated Circuits
CMOS	Complementary Metal Oxide Semiconductor
RRAM	Resistive Random Access Memory
HRS	High Resistance State
LRS	Low Resistance State
PCN	Pocket-Channel Framework
ZIF	Zeolitic Imidazolate Framework
BTC	Benzene-1mine-3-tricarboxylic acid
PET	Polyethylene Terephthalate
EGaIn	eutectic gallium-indium
GaInSn	Gallium-indium-tin
CP	Conductive Path
TE	Top Electrode
BE	Bottom Electrode
PVPy	Polyvinylpyrrolidone
PMMA	Poly-(methyl methacrylate)
PVA	Polyvinyl Alcohol
NAND	Not AND
NOR	Not OR
WORM	Write-Once-Read-Many
SCLC	Space Charge Limited Conduction
ECM	Electrochemical Metallization
CB	Conductive Bridge
I–V	Current Voltage Characteristic
STDP	Spike Timing Dependent Plasticity
SNN	Spiking Neural Network
MNIST	Mixed National Institute of Standards and Technology database
CNN	Convolutional Neural Networks

## References

[1] Chen C.L.P., Zhang C.Y. (2014). Data-Inntensive Applications, Challenges, Techniques and Tehnologies: A Surgey on Big Data. Inf. Sci..

[2] Zidan M.A., Strachan J.P., Lu W.D. (2018). The Future of Electronics Based on Memristive Systems. Nat. Electron..

[3] Reinsel D., Gantz J., Rydning J. (2018). The Digitization of the World from Edge to Core.

[4] Theis T., Wong H. (2017). The End of Moore’s Law: A New Beginning for Information Technology. Comput. Sci. Eng..

[5] Track E., Forbes N., Strawn G. (2017). The End of Moore’s Law. Comput. Sci. Eng..

[6] Patterson D., Anderson T., Cardwell N., Fromm R., Keeton K., Kozyrakis C., Thomas R., Yelick K. (1997). A Case for Intelligent RAM. IEEE Micro.

[7] Rogers B.M., Krishna A., Bell G.B., Jiang X.W. Scaling the Bandwidth Wall: Challenges in and Avenues for CMP Scaling. Proceedings of the 36th Annual International Symposium on Computer Architecture.

[8] Moore S. (2019). Another Step toward the End of Moore’s Law. IEEE Spectr..

[9] Strukov D.B., Snider G.S., Stewart D.R., Williams R.S. (2008). The Missing Memristor Found. Nature.

[10] Chua L. (1971). Memristor—The Missing Circuit Element. IEEE Trans. Circuit Theory.

[11] Chen Q.L., Zhang Y., Liu S.Z., Han T.T., Chen X.H., Xu Y.Q., Meng Z.Q., Zhang G.L., Zheng X.J., Zhao J.J. (2020). Switchable Perovskite Photovoltaic Sensors for Bioinspired Adaptive Machine Vision. Adv. Intell. Syst..

[12] Chen Q.L., Han T.T., Tang M.H., Zhang Z., Zheng X.J., Liu G. (2020). Improving the Recognition Accuracy of Memristive Neural Networks via Homogenized Analog Type Conductance Quantization. Micromachines.

[13] Chen Q.L., Liu G., Xue W.H., Shang J., Gao S., Yi X., Lu Y., Chen X.H., Tang M.H., Zheng X.J. (2019). Controlled Construction of Atomic Point Contact with 16 Quantized Conductance States in Oxide Resistive Switching Memory. ACS Appl. Electron. Mater..

[14] Ali K.A., Rizk M., Baghdadi A., Diguet J.P., Jomaah J. Crossbar Memory Architecture Performing Memristor Overwrite Logic. Proceedings of the 2019 26th IEEE International Conference on Electronics, Circuits and Systems (ICECS).

[15] Luo L., Dong Z.K., Duan S.K., Lai C.S. (2020). Memristorbased Stateful Logic Gates for Multi-Functional Logic Circuit. IET Circuits Devices Syst..

[16] Huo Q., Yang Y.M., Wang Y.M., Lei D.Y., Fu X.Q., Ren Q.R., Xu X.X., Luo Q., Xing G.Z., Chen C.Y. (2022). Computing-in-Memory Macro Based on Three-Dimensional Resistive Random-Access Memory. Nat. Electron..

[17] Wan W., Kubendran R., Schaefer C., Eryilmaz S.B., Zhang W.Q., Wu D., Deiss S., Raina P., Qian H., Gao B. (2022). A Compute-in-Memory Chip Based on Resistive Random-Access Memory. Nature.

[18] Zhang B., Chen W.L., Zeng J.M., Fan F., Gu J.W., Chen X.H., Yan L., Xie G.J., Liu S.Z., Yan Q. (2021). 90% Yield Production of Polymer Nanomemristor for In-Memory Computing. Nat. Commun..

[19] Kumar P., Zhu K., Gao X., Wang S.D., Lanza M., Thakur C.S. (2022). Hybrid Architecture Based on Two-Dimensional Memristor Crossbar Array and CMOS Integrated Circuit for Edge Computing. NPJ 2D Mater. Appl..

[20] Khan M., Kim J., Chougale M.Y., Furqan C.M., Saqib Q.M., Shaukat R.A., Kobayashi N.P., Mohammad B., Bae J., Kwok H. (2022). Ionic Liquid Multistate Resistive Switching Characteristics in Two Terminal Soft and Flexible Discrete Channels for Neuromorphic Computing. Microsyst. Nanoeng..

[21] Li Y., Zhang Z., Li J., Chen X.D., Kong Y., Wang F.D., Zhang G.X., Lu T.B., Zhang J. (2022). Low-Voltage Ultrafast Nonvolatile Memory Via Direct Charge Injection Through a Threshold Resistive-Switching Layer. Nat. Commun..

[22] Stiff-Roberts A., Ge W. (2017). Organic/Hybrid Thin Films Deposited by Matrix-Assisted Pulsed Laser Evaporation (Maple). Appl. Phys. Rev..

[23] Rullyani C., Ramesh M., Sung C. (2018). Natural Polymers for Disposable Organic Thin Film Transistors. Org. Electron..

[24] Kang J., Kim T., Hu S., Kim J., Kwak J.Y., Par J., Park J.K., Kim I., Lee S., Kim S. (2022). Cluster-Type Analogue Memristor by Engineering Redox Dynamics for High-Performance Neuromorphic Computing. Nat. Commun..

[25] Park S., Jeong H., Park J., Bae J., Choi S. (2022). Experimental Demonstration of Highly Reliable Dynamic Memristor for Artificial Neuron and Neuromorphic Computing. Nat. Commun..

[26] Sun K., Chen J., Yan X. (2021). The Future of Memristors: Materials Engineering and Neural Networks. Adv. Funct. Mater..

[27] Yuan L., Liu S.Z., Chen W.L. (2021). Organic Memory and Memristors: From Mechanisms, Materials to Devices. Adv. Mater..

[28] Lollar C., Qin J., Pang J., Yuan S., Becker B., Zhou H. (2018). Interior Decoration of Stable Metal-Organic Frameworks. Langmuir.

[29] Allendorf M., Schwartzberg A., Stavila V., Talin A.A. (2011). A Roadmap to Implementing Metal-Organic Frameworks in Electronic Devices: Challenges and Critical Directions. Eur. J..

[30] Stassen I., Burtch N., Talin A., Falcaro P., Allendorf M., Ameloot R. (2017). An Updated Roadmap for The Integration of Metal-Organic Frameworks with Electronic Devices and Chemical Sensors. Chem. Soc. Rev..

[31] Batten S., Champness N., Chen X. (2013). Terminology of Metal-Organic Frameworks and Coordination Polymers. Pure Appl. Chem..

[32] Takaishi S., Hosoda M., Kajiwara T., Miyasaka H., Yamashita M., Nakanishi Y., Kitagawa Y., Yamaguchi K., Kobayashi A., Kitagawa H. (2009). Electroconductive Porous Coordination Polymer Cu[Cu(pdt)_2_] Composed of Donor and Acceptor Building Units. Inorg. Chem..

[33] Kobayashi Y., Jacobs B., Allendorf M., Long J. (2010). Conductivity, Doping, and Redox Chemistry of a Microporous Dithiolene-Based Metal-Organic Framework. Chem. Mater..

[34] Alessandro D., Kanga J., Caddy J. (2011). Towards Conducting Metal-Organic Frameworks. Aust. J. Chem..

[35] Yoon S., Warren S., Grzybowski B. (2014). Storage of Electrical Information in Metal-Organic-Framework Memristors. Angew. Chem. Int. Ed..

[36] Feldblyum J., McCreery C., Andrews S., Kurosawa T. (2015). Few-Layer, Large-Area, 2D Covalent Organic Framework Semiconductor Thin Films. Chem. Commun..

[37] Xie L., Skorupskii G., Dinca M. (2020). Electrically Conductive Metal-Organic Frameworks. Chem. Rev..

[38] Chen A., Haddad S., Wu Y.C., Fang T.N., Lan Z., Avanzino S., Pangrle S., Buynoski M., Rathor M., Cai W. Non-volatile resistive switching for advanced memory applications. Proceedings of the International Electron Devices Meeting 2005 (IEDM) Technical Digest.

[39] Onlaor K., Thiwawong T., Tunhoo B. (2016). Electrical switching and conduction mechanisms of nonvolatile write-once-read-many-times memory devices with ZnO nanoparticles embedded in polyvinylpyrrolidone. Org. Electron..

[40] Wang Y., Lv Z., Liao Q., Shan H., Chen J., Zhou Y., Zhou L., Chen X., Roy V.A.L., Wang Z. (2018). Synergies of Electrochemical Metallization and Valance Change in All-Inorganic Perovskite Quantum Dots for Resistive Switching. Adv. Mater..

[41] Rehman M.M., Siddiqui G.U., Gul J.Z., Kim S.W., Lim J.H., Choi K.H. (2016). Resistive Switching in All-Printed, Flexible and Hybrid MoS_2_-PVA Nanocomposite based Memristive Device Fabricated by Reverse Offset. Sci. Rep..

[42] Qian K., Tay R.Y., Nguyen V.C., Wang J., Cai G., Chen T., Teo E.H.T., Lee P.S. (2016). Hexagonal Boron Nitride Thin Film for Flexible Resistive Memory Applications. Adv. Funct. Mater..

[43] Hosseini N.R., Lee J.S. (2015). Biocompatible and Flexible Chitosan-Based Resistive Switching Memory with Magnesium Electrodes. Adv. Funct. Mater..

[44] Huang Y.R., Lin X.L., Chen B., Zheng H.D., Chen Z.R., Li H.H., Zheng S.T. (2021). Thermal-Responsive Polyoxometalate–Metalloviologen Hybrid: Reversible Intermolecular Three-Component Reaction and Temperature-Regulated Resistive Switching Behaviors. Angew. Chem. Int. Ed..

[45] Gallegos I.R., Niño J.Á., Arriaga D.H., Reyes M.R., Sandoval R.L. (2017). Flexible Rewritable Organic Memory Devices Using Nitrogen-Doped CNTs/PEDOT: PSS Composites. Org. Electron..

[46] Cheng X.F., Shi E.B., Hou X., Shu J., He J.H., Li H., Xu Q.F., Li N.J., Chen D.Y., Lu J.M. (2017). 1D π-d Conjugated Coordination Polymers for Multilevel Memory of Long-Term and High-Temperature Stability. Adv. Electron. Mater..

[47] He K., Liu Y., Wang M., Chen G., Jiang Y., Yu J., Wan C., Qi D., Xiao M., Leow W.R. (2020). An Artificial Somatic Reflex Arc. Adv. Mater..

[48] Cavka J.H., Jakosen S., Olsbye U., Guillou N., Lamberti C., Bordiga S., Lillerud K.P. (2008). A New Zirconium Inorganic Building Brick Forming Metal Organic Frameworks with Exceptional Stability. J. Am. Chem. Soc..

[49] Liu Y., Wang H., Shi W.X., Zhang W.N., Yu J.C., Chandran B.K., Cui C.L., Zhu B., Liu Z.Y., Li B. (2016). Alcohol-Mediated Resistance-Switching Behavior in Metal-Organic Framework-Based Electronic Devices. Angew. Chem. Int. Ed..

[50] Yao Z.Z., Pan L., Liu L.Z., Zhang J.D., Lin Q.J., Ye Y.X., Zhang Z.J., Xiang S.C., Chen B.L. (2019). Simultaneous Implementation of Resistive Switching and Rectifying Effects in A Metal-Organic Framework with Switched Hydrogen Bond Pathway. Sci. Adv..

[51] Pan L., Liu G., Li H., Meng S., Han L., Shang J., Chen B., Prats A.E.P., Lu W., Zou X.D. (2014). A Resistance-Switchable and Ferroelectric Metal-Organic Framework. J. Am. Chem. Soc..

[52] Chen L., Gong Q., Chen Z. (2021). Preparation and Application of Ultra-Thin Two Dimensional MOF Nanomaterials. Prog. Chem..

[53] Ding G.L., Wang Y.X., Zhang G.X., Zhou K., Zeng K.L., Li Z.X., Zhou Y., Zhang C., Chen X.L., Han S.T. (2019). 2D Metal-Organic Framework Nanosheets with Time-Dependent and Multilevel Memristive Switching. Adv. Funct. Mater..

[54] Shekhah O., Wang H., Kowarik S., Schreiber F., Paulus M., Tolan M., Steremann C., Evers F., Zacher D., Fischer R.A. (2007). Step-by-Step Route for the Synthesis of Metal-Organic Frameworks. J. Am. Chem. Soc..

[55] Yi X.H., Yu Z., Niu X.H., Shang J., Mao G.Y., Yin T.H., Yang H.L., Xue W.H., Dhanapal P., Qu S.X. (2019). Intrinsically Stretchable Resistive Switching Memory Enabled by Combining a Liquid Metal-Based Soft Electrode and a Metal-Organic Framework Insulator. Adv. Electron. Mater..

[56] Albano L.G.S., Vello T.P., Camargo D.H.S.D., Silva R.M.L.D., Padilha A.C.M., Fazzio A., Bufon C.C.B. (2020). Ambipolar Resistive Switching in an Ultrathin Surface-Supported Metal-Organic Framework Vertical Heterojunction. Nano Lett..

[57] Wang J., Feng Y., Zhang B. (2022). MOF-COF Hybrid Frameworks Materials. Prog. Chem..

[58] Talin A., Centrone A., Ford A.C., Foster M.E., Stavila V., Haney P., Kinney R.A., Szalai V., Gabaly F.E., Yoon H.P. (2014). Tunable Electrical Conductivity in Metal-Organic Framework Thin-Film Devices. Science.

[59] Wang Z., Nminibapiel D., Shrestha P., Liu J., Guo W., Weidler P.G., Baumgart H., Wöll C., Redel E. (2016). Resistive Switching Nanodevices Based on Metal-Organic Frameworks. ChemNanoMat.

[60] Tran T.N.H., Le T.H., Ta H.K.T., Dang Y.T., Nguyen L.T.H., Doan T.H.L., Fang C., Hwang I., Phan T.B., Pham N.K. (2021). C-AFM Study on Multi-Resistive Switching Modes Observed in Metal-Organic Frameworks Thin Films. Org. Electron..

[61] Park S.S., Hontz E.R., Sun L., Hendon C.H., Walsh A., Voorhis T.V., Dincă M. (2015). Cation-Dependent Intrinsic Electrical Conductivity in Isostructural Tetrathiafulvalene-Based Microporous Metal-Organic Frameworks. J. Am. Chem. Soc..

[62] Chen D.S., Xing H.Z., Su Z.M., Wang C.G. (2016). Electrical Conductivity and Electroluminescence of a New Anthracene-Based Metal-Organic Framework With P-Conjugated Zigzag Chains. Chem. Commun..

[63] Lin K., Adhikari A.K., Ku C.N., Chiang C.L., Kuo H. (2012). Synthesis and Characterization of Porous HKUST-1 Metal Organic Frameworks for Hydrogen Storage. Int. J. Hydrogen Energy.

[64] Chui S.S.Y., Lo S.M.F., Charmant J.P.H., Orpen A.G., Williams I.D. (1999). A Chemically Functionalizable Nanoporous Material [Cu_3_(TMA)_2_(H_2_O)_3_]_n_. Science.

[65] Hayashi H., Côté A.P., Furukawa H., Keeffe M.O., Yaghi O.M. (2007). Zeolite A Imidazolate Frameworks. Nat. Mater..

[66] He Y., Huang Y.R., Li H.H., Chen Z.R., Jiang R. (2019). Encapsulating Halometallates into 3-D Lanthanide-Viologen Frameworks: Controllable Emissions, Reversible Thermochromism, Photocurrent Responses, and Electrical Bistability Behaviors. Inorg. Chem..

[67] Chen B., Huang Y., Song K., Lin X.L., Li H.H., Chen Z.R. (2021). Molecular Nonvolatile Memory Based On [α-GeW_12_O_49]_^4−^/Metalloviologen Hybrids Can Work at High Temperature Monitored by Chromism. Chem. Mater..

[68] Rana S., Prasoon A., Jha P.K., Prathamshetti A., Ballav N. (2017). Thermally Driven Resistive Switching in Solution-Processable Thin Films of Coordination Polymers. J. Phys. Chem. Lett..

[69] Zhao M.T., Wang Y.X., Ma Q.L., Huang Y., Zhang X., Ping J.F., Zhang Z.C., Lu Q.P., Yu Y.F., Xu H. (2015). Ultrathin 2D Metal-Organic Framework Nanosheets. Adv. Mater..

[70] Pan L., Ji Z., Yi X., Zhu X., Zhu X.J., Chen X.X., Shang J., Liu G., Li R.W. (2015). Metal-Organic Framework Nanofilm for Mechanically Flexible Information Storage Applications. Adv. Funct. Mater..

[71] Park M.J., Lee J.S. (2017). Zeolitic-Imidazole Framework Thin Film-Based Flexible Resistive Switching Memory. RSC Adv..

[72] Huang X., Zheng B., Liu Z., Tan C., Liu J.Q., Chen B., Li H., Chen J.Z., Zhang X., Fan Z.X. (2014). Coating Two-Dimensional Nanomaterials with Metal-Organic Frameworks. ACS Nano.

[73] Zheng N., Masel R.I. (2006). Rapid Production of Metal-Organic Frameworks Via Microwave-Assisted Solvothermal Synthesis. J. Am. Chem. Soc..

[74] Wang S.Z., McGuirk M., Andrea A., Mason J.A., Mirkin C.A. (2018). Metal-Organic Framework Nanoparticles. Adv. Mater..

[75] Zhang Z.P., Nie Y.J., Hua W.W., Xu J.X., Ban C.Y., Xiu F., Liu J.Q. (2020). Interfacial Synthesis of a Large-Area Coordination Polymer Membrane for Rewritable Nonvolatile Memory Devices. RSC Adv..

[76] Kutubi H.A., Gascon J., Sudhölter E.J.R., Rassaei L. (2015). Electrosynthesis of Metal–Organic Frameworks: Challenges and Opportunities. ChemElectroChem.

[77] Li W.J., Lü J., Gao S.Y., Li Q.H., Cao R. (2014). Electrochemical preparation of metal–organic framework films for fast detection of nitro explosives. J. Mater. Chem. A.

[78] Alizadeh S., Nematollahi D. (2019). Convergent and Divergent Paired Electrodeposition of Metal-Organic Framework Thin Films. Sci. Rep..

[79] Liu L., Dong J., Liu J., Liang Q., Song Y.R., Li W.H., Lei S.B., Hu W.P. (2021). High-quality Two-Dimensional Metal-Organic Framework Nanofilms for Nonvolatile Memristive Switching. Small Struct..

[80] Sun L.F., Zhang Y.S., Han G., Hwang G., Jiang J.B., Joo B., Watanabe K., Taniguchi T., Kim Y.M., Yu W.J. (2019). Self-Selective Van Der Waals Heterostructures for Large Scale Memory Array. Nat. Commun..

[81] Vu Q.A., Kim H., Nguyen V.L., Won U.Y., Adhikari S., Kim K., Lee Y.H., Yu W.J. (2017). A High-On/Off-Ratio Floating-Gate Memristor Array on A Flexible Substrate Via CVD-Grown Large-Area 2D Layer Stacking. Adv. Mater..

[82] Yu Z., Shang J., Niu X.H., Liu Y.W., Liu G., Dhanapal P., Zheng Y.N., Yang H.L., Wu Y.Z., Zhou Y.L. (2018). A Composite Elastic Conductor with High Dynamic Stability Based on 3D-Calabash Bunch Conductive Network Structure for Wearable Devices. Adv. Electron. Mater..

[83] Yu Z., Ying W.B., Pravarthanaac D., Li Y.Y., Mao G.Y., Liu Y.W., Hu C., Zhang W.X., He P.X., Zhong Z.C. (2020). Stretchable tactile sensor with high sensitivity and dynamic stability based on vertically aligned urchin-shaped nanoparticles. Mater. Today Phys..

[84] Lee B.H., Dong L.I., Bae H., Seong H., Jeon S.B., Seol M.L., Han J.W., Meyyappan M., Im S.G., Choi Y.K. (2016). Foldable and Disposable Memory on Paper. Sci. Rep..

[85] Kulachenkov N., Haar Q., Yankin A., Pierson J.F., Nominé A., Milichko V.A. (2021). MOF-Based Sustainable Memory Devices. Adv. Funct. Mater..

[86] Prezioso M., Merrikh-Bayat F., Hoskins B.D., Adam G.C., Likharev K.K., Stukov D.B. (2015). Training and Operation of an Integrated Neuromorphic Network Based on Metal-Oxide Memristors. Nature.

[87] Li Z.W., Chen P.Y., Xu H., Yu S.M. (2017). Design of Ternary Neural Nnetwork with 3-D Vertical RRAM Array. IEEE Trans. Electron. Devices.

[88] Kim B., Li H. Leveraging 3D Vertical RRAM to Developing Neuromorphic Architecture for Pattern Classification. Proceedings of the 2020 IEEE Computer Society Annual Symposium on VLSI (ISVLSI).

[89] Xiao T.P., Bennett C.H., Feinberg B., Agarwal S., Marinella M.J. (2020). Analog Architectures for Neural Network Acceleration Based on Non-Volatile Memory. Appl. Phys. Rev..

[90] Wang I.T., Lin Y.C., Wang Y.F., Hsu C.W., Hou T.H. 3D Synaptic Architecture with Ultralow Sub-10 fJ Energy Per Spike for Neuromorphic Computation. Proceedings of the 2014 IEEE International Electron Devices Meeting.

[91] Gao B., Bi Y.J., Chen H.Y., Liu R., Huang P., Chen B., Liu L.F., Liu X.Y., Yu S.M., Wong H.S.P. (2014). Ultra-Low-Energy Three-Dimensional Oxide-Based Electronic Synapses for Implementation of Robust High-Accuracy Neuromorphic Computation Systems. ACS Nano.

[92] Li H.T., Li K.S., Lin C.H., Hsu J.L., Chiu W.C., Chen M.C., Wu T.T., Sohn J., Eryilmaz S.B., Shieh J.M. Four-Layer 3D Vertical RRAM Integrated with FinFET as a Versatile Computing Unit for Brain-Inspired Cognitive Information Processing. Proceedings of the 2016 IEEE Symposium on VLSI Technology.

[93] Lin P., Li C., Wang Z.R., Li Y.N., Jiang H., Song W.H., Rao M.Y., Zhuo Y., Upadhyay N.K., Barnell M. (2020). Three-Dimensional Memristor Circuits as Complex Neural Networks. Nat. Electron..

[94] Xie P.S., Huang Y.L., Wang W., Meng Y., Lai Z., Wang F., Yip S.P., Bu X., Wang W.J., Li D.J. (2022). Ferroelectric P (VDF-Trfe) Wrapped Ingaas Nanowires for Ultralow-Power Artificial Synapses. Nano Energy.

[95] Ni Y., Feng J.L., Liu J.Q., Yu H., Wei H.H., Du Y., Liu L., Sun L., Zhou J.L., Xu W.T. (2022). An Artificial Nerve Capable of UV-Perception, NIR-Vis Switchable Plasticity Modulation, and Motion State Monitoring. Adv. Sci..

[96] Kim S.W., Jeong Y.J., Bidenko P., Lim H.R., Jeon Y.R., Kim H., Lee Y.J., Geum D.M., Han J.H., Choi C. (2020). 3D Stackable Synaptic Transistor for 3D Integrated Artificial Neural Networks. ACS Appl. Mater. Interfaces.

[97] Choi Y., Oh S., Qian C., Park J.H., Cho J.H. (2020). Vertical Organic Synapse Expandable to 3D Crossbar Array. Nat. Commun..

[98] Lian H., Cheng X.Z., Hao H.T., Han J.B., Lau M.T., Li Z., Zhou Z., Dong Q.C., Wong W.Y. (2022). Metal-Containing Organic Compounds for Memory and Data Storage Applications. Chem. Soc. Rev..

[99] Wang Y., Yang R., Deng Q., Fan C., Zhang S., Yan Y. (2022). Application of Bimetallic MOFs and Their Derivatives in Electrochemical Energy Storage. Prog. Chem..

[100] Xie L.H., Ling Q.D., Hou X.Y., Huang W. (2008). An Effective Friedel-Crafts Postfunctionalization of Poly(N-vinylcarbazole) to Tune Carrier Transportation of Supramolecular Organic Semiconductors Based on π-Stacked Polymers for Nonvolatile Flash Memory Cell. J. Am. Chem. Soc..

[101] Han S.B., Warren S.C., Yoon S.M., Malliakas C.D., Hou X.L., Wei Y.H., Kanatzidis M.G., Grzybowski B.A. (2015). Tunneling Electrical Connection to the Interior of Metal-Organic Frameworks. J. Am. Chem. Soc..

[102] Ding G.L., Yang B.D., Zhou K., Zhang C., Wang Y.X., Yang J.Q., Han S.T., Zhai Y.B., Roy V.A.L., Zhou Y. (2020). Synaptic Plasticity and Filtering Emulated in Metal-Organic Frameworks Nanosheets Based Transistors. Adv. Electron. Mater..

[103] Jeon Y.J., An H., Kim Y., Jeon Y.P., Kim T.W. (2021). Highly Reliable Memristive Devices with Synaptic Behavior Via Facilitating Ion Transport of The Zeolitic Imidazolate Framework-8 Embedded into A Polyvinylpyrrolidone Polymer Matrix. Appl. Surf. Sci..

[104] Liu Y.X., Wei Y.N., Liu M.H., Bai Y.C., Liu G.C., Wang X.Y., Shang S.C., Gao W.Q., Du C.S., Chen J.Y. (2021). Two-Dimensional Metal-Organic Framework Film for Realizing Optoelectronic Synaptic Plasticity. Angew. Chem. Int. Ed..

[105] Liu S.Z., Chen X.H., Liu G. (2020). Conjugated Polymers for Information Storage and Neuromorphic Computing. Polym. Int..

[106] Chen Q.L., Han T.T., Zeng J.M., He Z.L., Liu Y.L., Sun J.L., Tang M.H., Zhang Z., Gao P.Q., Liu G. (2022). Perovskite-Based Memristor with 50-Fold Switchable Photosensitivity for In-Sensor Computing Neural Network. Nanomaterials.

[107] Li C., Belkin D., Li Y.N., Hu M., Ge N., Jiang H., Montgomery E., Lin P., Wang Z.R., Song W.H. (2018). Efficient and Self-Adaptive In-Situ Learning in Multilayer Memristor Neural Networks. Nat. Commun..

[108] Souri Z., Ardakani M.M., Alizadeh S., Nematollahi D. (2022). Template-free Electrodeposition of Sponge-like Porous Polymer Interwoven with the Bi-Metallic Metal-Organic Framework and Reduced Graphene Oxide and Application in Energy Storage Device. J. Energy Storage.

[109] Rahimpoor R., Firoozichahak A., Nematollahi D., Alizadeh S., Alizadeh P.M., Langari A.A.A. (2021). Bio-monitoring of Non-Metabolized BTEX Compounds in Urine by Dynamic Headspace-Needle Trap Device Packed with 3D Ni/Co-BTC Bimetallic Metal-Organic Framework as an Efficient Absorbent. Microchem. J..

